# Drr4covid: Learning Automated COVID-19 Infection Segmentation From Digitally Reconstructed Radiographs

**DOI:** 10.1109/ACCESS.2020.3038279

**Published:** 2020-11-16

**Authors:** Pengyi Zhang, Yunxin Zhong, Yulin Deng, Xiaoying Tang, Xiaoqiong Li

**Affiliations:** 1 School of Life Science, Beijing Institute of Technology Beijing 100081 China; 2 Key Laboratory of Convergence Medical Engineering System and Healthcare TechnologyMinistry of Industry and Information Technology Beijing 100081 China

**Keywords:** COVID-19~diagnosis, infection segmentation, DRRs, X-ray imaging, deep learning

## Abstract

Automated infection measurement and COVID-19 diagnosis based on Chest X-ray (CXR) imaging is important for faster examination, where infection segmentation is an essential step for assessment and quantification. However, due to the heterogeneity of X-ray imaging and the difficulty of annotating infected regions precisely, learning automated infection segmentation on CXRs remains a challenging task. We propose a novel approach, called DRR4Covid, to learn COVID-19 infection segmentation on CXRs from digitally reconstructed radiographs (DRRs). DRR4Covid consists of an infection-aware DRR generator, a segmentation network, and a domain adaptation module. Given a labeled Computed Tomography scan, the infection-aware DRR generator can produce infection-aware DRRs with pixel-level annotations of infected regions for training the segmentation network. The domain adaptation module is designed to enable the segmentation network trained on DRRs to generalize to CXRs. The statistical analyses made on experiment results have indicated that our infection-aware DRRs are significantly better than standard DRRs in learning COVID-19 infection segmentation (p < 0.05) and the domain adaptation module can improve the infection segmentation performance on CXRs significantly (p < 0.05). Without using any annotations of CXRs, our network has achieved a classification score of (Accuracy: 0.949, AUC: 0.987, F1-score: 0.947) and a segmentation score of (Accuracy: 0.956, AUC: 0.980, F1-score: 0.955) on a test set with 558 normal cases and 558 positive cases. Besides, by adjusting the strength of radiological signs of COVID-19 infection in infection-aware DRRs, we estimate the detection limit of X-ray imaging in detecting COVID-19 infection. The estimated detection limit, measured by the percent volume of the lung that is infected by COVID-19, is 19.43% ± 16.29%, and the estimated lower bound of infected voxel contribution rate for significant radiological signs of COVID-19 infection is 20.0%. Our codes are made publicly available at https://github.com/PengyiZhang/DRR4Covid.

## I. Introduction

The highly contagious Coronavirus Disease 2019 (COVID-19), caused by the severe acute respiratory syndrome coronavirus 2 (SARS-CoV-2) virus [1]–[3], has spread rapidly to most countries in the world. Globally, as of 2:34pm CEST, 7 July 2020, there have been 11,500,302 confirmed cases of COVID-19, including 535,759 deaths, reported to World Health Organization (WHO) [4]. Rapid detection and confirmation of COVID-19 infection is critical to prevent the spread of this epidemic.

Radiological imaging, such as Computed Tomography (CT) and Chest X-ray (CXR), is currently used to provide visual evidence for confirming COVID-19 positive patients in clinical practice. CT scan provides accurate 3D images of the lungs that are able to detect very small lesions effectively such as lung nodule and tumor. However, the workflow of CT imaging, involving several pre-scan events [5], is relatively complex, and meanwhile, CT examinations are costly. As the number of infected patients rapidly increases, the routine use of CT brings heavy burden to the radiology department [6]. In contrast, CXR examination is much easier, faster and less costly, and provides high-resolution 2D images of the lungs that can detect a variety of lung conditions such as pneumonia, emphysema and cancer. CXRs are the typical first-line imaging modality used for patients under investigation of COVID-19 [7]. Therefore, automated infection measurement and COVID-19 diagnosis based on CXRs is important for faster examination, where infection segmentation is an essential step for assessment and quantification.

Many approaches have been proposed for automated COVID-19 diagnosis based on CXRs, and have claimed notable detection accuracy of COVID-19 infection. However, due to the lack of sufficient CXRs with pixel-level annotations of infected regions, the majority of these approaches are designed by using classification models rather than segmentation models. The projective nature of X-ray imaging causes large overlapping of anatomies, fuzzy object boundaries and complex texture patterns, thus making it extremely difficult to delineate infected regions precisely on CXRs even for experienced clinicians [8]. As an alternative, some researchers have leveraged the interpretability of classification model (e.g., saliency map or attention map) to locate the infected regions roughly. However, such methods are unable to produce accurate COVID-19 infection segmentation for further assessment and quantification. Currently, to our best knowledge, no effective approaches have been developed for automated COVID-19 infection segmentation on CXRs as reviewed by Shen *et al.* [7].

Digitally reconstructed radiograph (DRR) [9]–[12] is a synthetic X-ray image that is generated by simulating the passage of X-rays through a 3D CT volume in specific poses (position and orientation) within a virtual imaging system. CXR findings of COVID-19 infection reflect those described by CT [13] such as bilateral, peripheral consolidation and/or ground glass opacities (GGOs) [7], [14], [15]. Besides, delineating infected regions in 3D CT scans is much easier than in heterogeneous 2D CXRs because CT scans can provide accurate 3D images of the lungs rather than heterogeneous 2D images. Thus, we propose to learn automated COVID-19 infection segmentation on CXRs from labeled DRRs by leveraging the publicly available CT scans with voxel-level annotations of infected regions and the correlation between DRRs and CXRs.

To this end, we propose a novel approach, called DRR4Covid, which can learn automated COVID-19 infection segmentation on CXRs from labeled DRRs. We design DRR4Covid with a modular framework, which consists of an infection-aware DRR generator, a deep segmentation network, and a domain adaptation module. Given a CT volume with voxel-level infection annotations, our infection-aware DRR generator can produce DRRs with adjustable radiological signs of COVID-19 infection, and generate pixel-level annotations of infected regions that match the DRRs accurately. Although such synthetic DRRs are photo-realistic, there is still a gap between synthetic DRRs and real CXRs, which may lead to a poor segmentation performance on real CXRs. Therefore, we introduce a domain adaptation module to train networks on labeled DRRs and unlabeled CXRs together. In this article, we provide a simple but effective implementation of DRR4Covid by using a domain adaptation module based on Maximum Mean Discrepancy (MMD), and a FCN-based [16] network with a classification header and a segmentation header. Extensive experiment results have confirmed the efficacy of our method; specifically, without using any annotations of CXRs, our network has achieved a classification score of (Accuracy: 0.949, AUC: 0.987, F1-score: 0.947) and a segmentation score of (Accuracy: 0.956, AUC: 0.980, F1-score: 0.955) on a test set with 558 normal cases and 558 positive cases. Besides, by adjusting the strength of radiological sign of COVID-19 infection in synthetic DRRs, we estimate the detection limit of X-ray imaging in detecting COVID-19 infection. The estimated detection limit, measured by the percent volume of the lung that is infected by COVID-19, is 19.43% ± 16.29%, and the estimated lower bound of the contribution rate of infected voxels for significant radiological signs of COVID-19 infection is 20.0%.

The novelties and contributions of our study mainly come from four major aspects:

1) We propose a novel approach, i.e., DRR4Covid, to learn automated COVID-19 infection segmentation on CXRs. To our best knowledge, this is the first attempt to learn automated COVID-19 infection segmentation on CXRs by using the labeled DRRs that are generated from Chest CT scans. Owing to the modular framework, our DRR4Covid can be implemented flexibly with the off-the-shelf segmentation networks and domain adaptation algorithms. Moreover, DRR4Covid is a unified approach that can be applied to other lesion segmentation (e.g., lung nodule and tumor) on X-ray images;
2) We design an infection-aware DRR generator to synthesize infection-aware DRRs with pixel-level annotations of infected regions for training segmentation network. The statistical analyses made on experiment results have confirmed that our infection-aware DRRs are significantly better than standard DRRs in learning COVID-19 infection segmentation (p < 0.05);
3) We provide a simple but effective implementation of DRR4Covid by using a domain adaptation module based on Maximum Mean Discrepancy (MMD), and a FCN-based network with a classification header and a segmentation header. The statistical analyses made on experiment results have confirmed that the domain adaptation module can improve the infection segmentation performance on CXRs significantly (p < 0.05);
4) We estimate the detection limit of X-ray imaging in detecting COVID-19 infection for the first time, which is of great significance for the severity assessment of COVID-19 infection based on X-ray imaging.

## II. Related Work

In this section, we review the related work from three aspects, including DRR, domain adaptation and CXR based screening of COVID-19 in light of infection segmentation.

### A. DRR

A digitally reconstructed radiograph (DRR) [9]–[12] is a synthetic X-ray image that is generated by simulating the passage of X-rays through a 3D CT volume in specific poses (position and orientation) within a virtual imaging system. DRRs are generally used as reference images by the intensity based 2D to 3D image registration algorithms to verify the correct setup position of a patient for many radiotherapy treatments [17]–[19]. Each pixel value of DRR is obtained by calculating the radiological path length (RPL) [20], i.e., the summation of the length travelled by the ray in each voxel, multiplied by the relative CT intensity of the voxel that is measured in Hounsfield units (HUs). Thus, with a high complexity level of $\rm {O}(n^{3})$, the synthesis of DRRs is computationally intensive by nature [21]. Meanwhile, in the iterative optimization of 2D–3D image registration algorithms, the synthesis of DRRs is usually performed many times to calculate the similarity measure [19], which greatly limits the running speed of 2D-3D image registration algorithms [12]. Therefore, the majority of previous studies have focused on this problem and have proposed plenty of improved approaches to accelerate the synthesis of DRRs [11], [12], [19]–[23]. In contrast, we are more concerned with the consistency between DRRs and the infection annotation masks. Thus, we directly design our infection-aware DRR generator based on SiddonGpuPy [24], which combines the serial algorithm proposed by Jacob [11] to improve the original Siddon’s algorithm [9], and the parallel implementation proposed by Greef *et al.* [20].

The most closely related work is TD-GAN [8] and DeepDRR [25], [26]. TD-GAN aims to learn automatic parsing of anatomical objects in X-ray images from labeled 3D CT scans by using synthetic labeled DRRs. The pixel-level annotations of anatomical objects are obtained by projecting 3D CT labels along the same trajectories used in the synthesis of DRRs. TD-GAN adopts the CycleGAN architecture to perform unpaired image-to-image translation and unsupervised domain adaptation to enable the segmentation models trained on DRRs to generalize to real X-ray images. Similar strategy is also used by X2CT-GAN [27] to reduce the gap between synthetic DRRs and real X-ray images. Unlike TD-GAN and X2CT-GAN, DeepDRR attempts to produce more realistic radiographs and fluoroscopy from 3D CT scans to enable machine learning models trained directly on DeepDRRs to generalize to clinical data without the need for domain adaptation. DeepDRR has been used in anatomical landmark detection in pelvic X-ray and to simulate X-rays of the femur during insertion of dexterous manipulators in orthopedic surgery. Both TD-GAN and DeepDRR care more about the anatomical structures than the lesion regions. Given a CT with COVID-19 infection, the existing DRR generators may produce a DRR with no findings due to the heterogeneity of DRRs. It is tough to keep the consistency between standard DRRs and annotation masks of lesion regions by using existing DRR generators. Therefore, we design a new infection-aware DRR generator to solve this problem through a category-weighted projection and RPL threshold method.

### B. Domain Adaptation

Domain adaptation aims at rectifying the distribution discrepancy between the training samples (source domain) and test samples (target domain) [28] and tuning the model toward better generalization onto the target domain in a supervised or unsupervised manner. Numerous domain adaptation methods have been proposed based on deep models recently as deep networks can learn more transferable features for domain adaptation and achieve better performance [29]–[31]. The main insight behind these approaches is to extract domain-invariant representations by embedding domain adaptation modules in the pipeline of deep learning [28], [32]–[38].

Existing deep domain adaptation methods align the distributions of source domain and target domain mainly from three perspectives. The first stream is image alignment, and image-to-image translation models are typically used to reduce the gap between source domain images and target domain images [8]. The second stream is feature alignment [32]–[37], which is the mainstream approach and aims to learn domain-invariant deep features. The last stream is output alignment, which is often used to learn semantic segmentation of urban scenes from synthetic data [28], [38]. Moreover, we recognize that there are two main approaches to perform feature alignment, including adversarial approach [34], [35], [39]–[41] and non-adversarial approach [31], [33], [36], [42]–[44]. The adversarial approach motivates deep models to extract domain-invariant features through adversarial training. It is done by training task-specific deep models to minimize the task-specific loss and the adversarial loss simultaneously, thereby fooling the domain discriminator to maximize the probability of deep features from source domain being classified as target domain. The non-adversarial approach is statistic moment matching-based approach, involving maximum mean discrepancy (MMD) [33], [42], [43], central moment discrepancy (CMD) [44] and second-order statistics matching [36]. The statistic moment matching-based approach encourages deep models to extract domain-invariant deep features by minimizing the distance between the statistic moments of deep features from source domain and from target domain. MMD [45] is the most representative method, and has been widely used to measure the discrepancy between the source domain and target domain distributions [31]. Compared with the adversarial approaches, MMD-based methods are simple, stable and are easy to implement, and thus can facilitate to verify the efficacy of DRR4Covid quickly. In our implementation of DRR4Covid, we directly use an off-the-shelf MMD-based domain adaptation approach, i.e., LMMD proposed by Zhu *et al.* [31], to enable the deep models trained on DRRs to generalize to real CXRs.

### C. CXR Based Screening of COVID-19 in a View of Infection Segmentation

Segmentation is an essential step in automated infection measurement and COVID-19 diagnosis, which can provide the delineation of the regions of interest (ROIs), e.g., infected regions, in the CXRs for further assessment and quantification. Many approaches have been proposed for automated COVID-19 diagnosis based on CXRs. However, the majority of these approaches are based on classification models rather than segmentation models as reviewed by Shen *et al.* in [7] due to the aforementioned reasons. Some researchers have leveraged the interpretability of deep classification models to highlight the infected regions rather than accurately segmenting the infected regions. Specifically, Oh *et al.* [6] introduce a probabilistic Grad-CAM saliency map to indicate the multifocal lesions within CXRs in their local patch-based deep classification models for COVID-19 diagnosis. Such method is derived from a famous explanation technique, i.e., gradient weighted class activation map (Grad-CAM), and can effectively locate the radiological signs of COVID-19 infection, such as the multifocal ground-glass opacification and consolidations. Similarly, Karim *et al.* [46] use a revised Grad-CAM, i.e., Grad-CAM++, and layer-wise relevance propagation (LRP) [47] in classifying CXRs as Normal, Pneumonia and COVID-19 to indicate the class-discriminating regions in CXRs. Besides, Tabik *et al.* [48] adopt multiple explanation techniques, including occlusion [49], saliency [50], input X gradient [51], guided backpropagation [52], integrated gradients [53], and DeepLIFT [54], to investigate the interpretability of deep classification models and highlight the relevant infected regions of pneumonia and COVID-19 separately. To sum up, these approaches based on explanation techniques are mainly used for the inspection of deep models’ decision, and may not be suitable for further assessment and quantification. In comparison, our DRR4Covid is able to train deep segmentation models for precise infection segmentation directly without the need for the pixel-level infection annotations of real CXRs.

## III. Methods

In this section, we describe the modular framework of proposed DRR4Covid, and analyze the critical elements in the design of DRR4Covid, followed by an introduction of our implementation of DRR4Covid.

### A. Modular Framework of DRR4Covid

**FIGURE 1.**
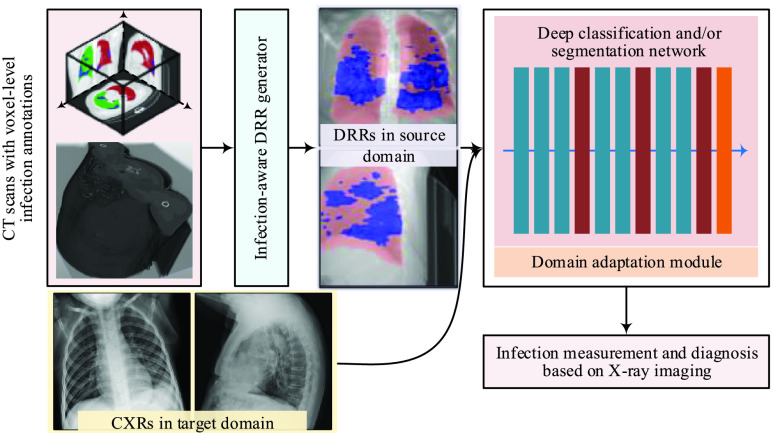
The modular framework of proposed DRR4Covid.

Given CT scans with voxel-level infection annotations and unlabeled CXRs, we aim to learn deep models to perform automated COVID-19 infection segmentation on CXRs. We design DRR4Covid with a modular framework as shown in Fig. 1. DRR4Covid consists of three key components, i.e., an infection-aware DRR generator, a deep classification and/or segmentation model, and a domain adaptation module. The basic workflow of DRR4Covid involves generating DRRs with pixel-level infection annotations from CT scans, and training deep models on synthetic labeled DRRs and unlabeled CXRs by using the domain adaptation module.

#### 1) Generating Labeled DRRs

The DRR generator is responsible for synthesizing photo-realistic DRRs that resemble real CXRs as much as possible and producing pixel-level infection annotations that match the DRRs precisely by projecting 3D CT annotations along the same trajectories used in synthesizing DRRs. High-quality labeled DRRs in the context of this article can be defined by two conditions. One is a good consistency between DRRs and infection annotation masks; the other one is a good correlation between the radiological signs of COVID-19 infection in DRRs and in real CXRs. As CXRs are typically considered less sensitive than 3D CT scans [7], it may happen that CT examination detects an abnormality, whereas the X-ray screening on the same patient reports no findings. DRRs also suffer from such problem, which will lead to the inconsistency between DRRs and infection annotation masks. This is the first key point for designing a high-quality DRR generator. The second key point is the correlation between the radiological signs of COVID-19 infection in real CXRs and in synthetic DRRs. Note that the synthetic DRRs and infection annotation masks are used later to train deep segmentation models. Thus, a large gap between the radiological signs of COVID-19 infection in real CXRs and in synthetic DRRs will make deep models trained on DRRs fail to generalize to real CXRs even if the domain adaptation module is applied.

#### 2) Training Deep Models With the Domain Adaptation Module

Although synthetic DRRs are photo-realistic, there is still a gap between DRRs and real CXRs. Thus, we introduce the domain adaptation module into the framework of DRR4Covid. According to the quality of synthetic labeled DRRs, the problem of training deep models on labeled DRRs and unlabeled CXRs for infection segmentation on CXRs by using the domain adaptation module can be divided into two categories. One is deep domain adaptation with fully supervised learning in the source domain (i.e., synthetic DRRs) and unsupervised learning in the target domain (i.e., real CXRs); the other one is deep domain adaptation with weakly supervised learning in the source domain and unsupervised learning in the target domain. The condition of the first category is a good consistency between DRRs and infection annotation masks. If such condition is not well satisfied, the problem will be turned into the second category due to the inaccurate synthetic infection annotations. Compared with the second one, the first category of problem is well defined, and has been extensively studied. In this article, we mainly focus on solving the first category of problem. Thus, we first implement a high-quality DRR generator, i.e., the infection-aware DRR generator.

### B. Infection-Aware DRR Generator

**FIGURE 2.**
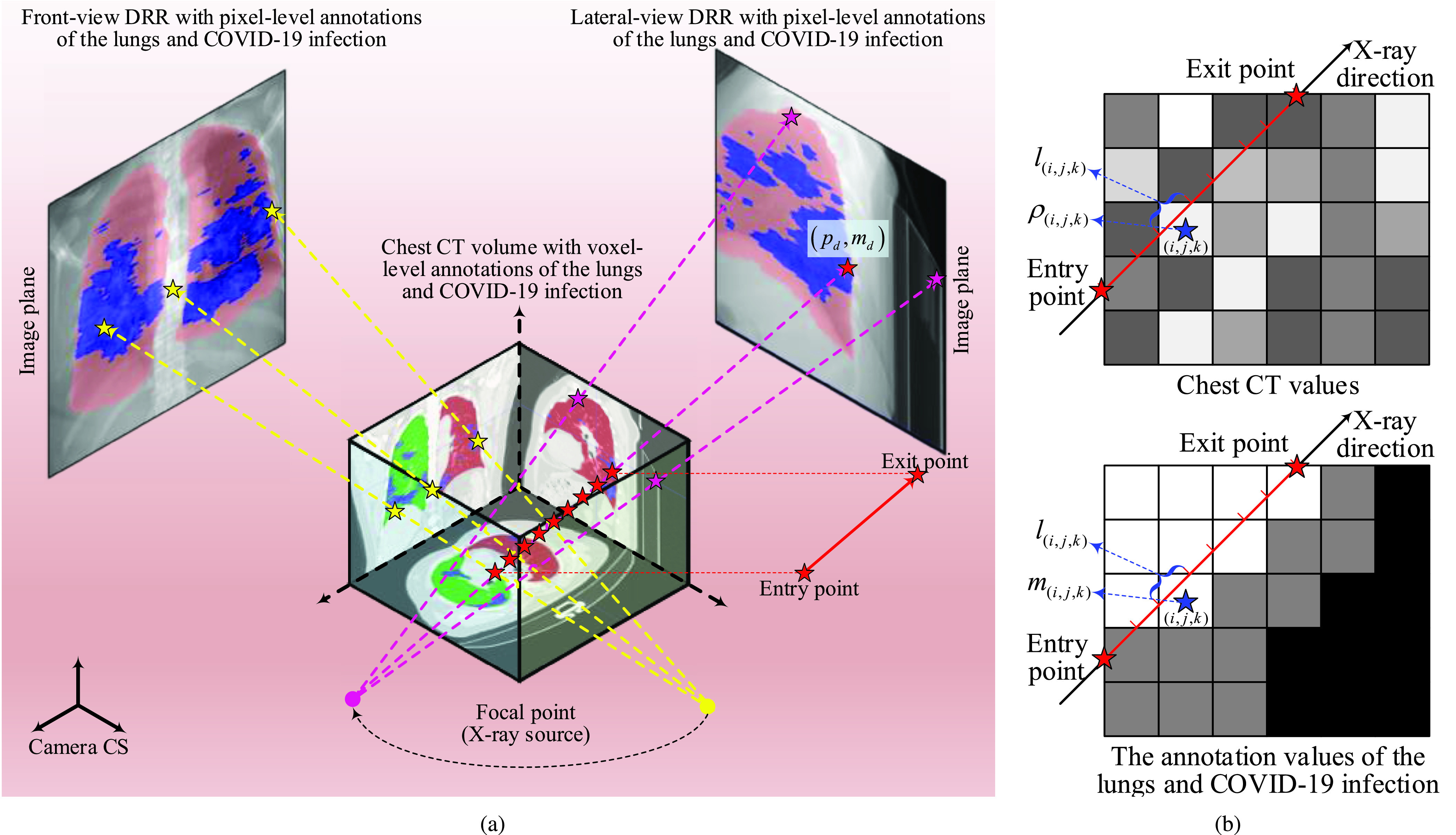
Illustration of the synthesis of infection-aware DRRs.

We design the infection-aware DRR generator to produce high-quality DRRs as defined in Section III-A. The standard DRR generator takes a CT volume or an infection annotation volume in a specific pose (position and orientation) as input and outputs a DRR or an infection mask. In contrast, our DRR generator takes both a CT volume and its infection annotation volume as input and produce a labeled DRR as illustrated in Fig. 2. A ray is casted from the X-ray source through labeled CT volumes to the center of each pixel of DRR. Each pixel value of DRR is obtained by calculating the class-weighted RPL [20], i.e., the class-weighted summation of the length travelled by this ray within each voxel, multiplied by the relative CT intensity of the voxel that is measured in HUs. The calculation of the ${d}$-th pixel of DRR $p_{d}$ is formulated as

\begin{equation*} p_{d} = \frac {\sum _{(i,j,k)\in \Omega _{d}}l_{(i,j,k)}\rho _{(i,j,k)}w_{(i,j,k)}}{\sum _{(i,j,k)\in \Omega _{d}}w_{(i,j,k)}/|\Omega _{d}|}\tag{1}\end{equation*}

where $\Omega _{d}$ is the 3D index set of the voxels in the X-ray direction, $|\Omega _{d}|$ is the number of voxels in $\Omega _{d}$, $l_{(i,j,k)}$ represents the normalized length travelled by the ray within the $(i,j,k)$-th voxel, $\rho _{(i,j,k)}$ and $w_{(i,j,k)}$ denote the CT value and the weight of the $(i,j,k)$-th voxel, respectively. The weight of the $(i,j,k)$-th voxel is defined as

\begin{align*} w_{(i,j,k)}= \begin{cases} w_{2}, & {\mathrm{if}}~m_{(i,j,k)}=2 \\ w_{1}, & {\mathrm{elif}}~m_{(i,j,k)}=1 \\ w_{0}, & {\mathrm{otherwise}} \end{cases}\Bigg |m_{(i,j,k)}\in \{0,1,2\}\tag{2}\end{align*}

where $m_{(i,j,k)}\in \{0,1,2\}$ is the category of the $(i,j,k)$-th voxel, 0, 1 and 2 represent the background, lungs and COVID-19 infection respectively. Note that the infection-aware DRR generator will produce standard DRRs when the weights of all categories are equal. On the other hand, the label of $p_{d}$, $m_{d}$, is computed as

\begin{align*} m_{d}= \begin{cases} 2, & {\mathrm{if}}~\pi ^{2}_{d}>T^{2} \\ 1, & {\mathrm{elif}}~\pi ^{2}_{d}\leq T^{2}~{\mathrm{and}}~\pi ^{1}_{d}>T^{1} \\ 0, & {\mathrm{otherwise}} \end{cases}\tag{3}\end{align*}

where $\pi ^{c}_{d}$ denotes the contribution rate of the voxels of category $c$ in calculating $p_{d}$, and $T^{c}$ represents the contribution threshold of category $c$. Specifically, $\pi ^{c}_{d}$ is defined as

\begin{equation*} \pi ^{c}_{d}=\frac {\sum _{(i,j,k)\in \Omega ^{c}_{d}}l_{(i,j,k)}w_{(i,j,k)}}{\sum _{(i,j,k)\in \Omega _{d}}l_{(i,j,k)}w_{(i,j,k)}}\qquad \Bigg |c\in \{0,1,2\}\tag{4}\end{equation*}

where $\Omega ^{c}_{d}$ denotes the 3D index set of the voxels of category $c$ in the X-ray direction.

The strength of the radiological signs of COVID-19 infection in CXRs and DRRs depends on the contribution rate of infected voxels (CRIV) due to the projective nature of X-ray imaging. A higher value of CRIV represents a larger number of infected voxels appear in the X-ray direction, and the radiological signs of COVID-19 infection, e.g., GGOs, will be more significant. Such property of X-ray imaging can be well modeled in “1” and “4” by our infection-aware DRR generator. Increasing the weight of infected voxels will improve the value of CRIV and vice versa. Accordingly, our infection-aware DRR generator can produce DRRs with different strengths of radiological signs of COVID-19 infection simply by adjusting the weight of infected voxel. The synthetic pixel-level annotations of COVID-19 infection are also computed based on the CRIV. Therefore, our infection-aware DRR generator can maintain the consistency between synthetic DRRs and infection annotation masks easily by increasing the weight of infected voxels when the value of CRIV is too small. To sum up, our infection-aware DRR generator has the following advantages:

1) By setting the weight of infected voxels to a very small value, our infection-aware DRR generator is able to produce DRRs with no findings, which are essential for training deep classification models for COVID-19 diagnosis;
2) By setting the weight of infected voxels to a relatively large value, our infection-aware DRR generator can generate high-quality DRRs with pixel-level annotations of infected regions, which are essential for training deep segmentation models for precise COVID-19 infection segmentation;
3) By adjusting the weight of infected voxels from small values to large values, our infection-aware DRR generator will synthesize a serial of labeled DRRs with different strengths of the radiological signs of COVID-19 infection. Such DRRs might be able to be used to estimate the detection limit of X-ray imaging in detecting COVID-19 infection.

### C. FCN-Based Network Equipped With a MMD-Based Domain Adaptation Module

#### 1) Network Architectures

**FIGURE 3.**
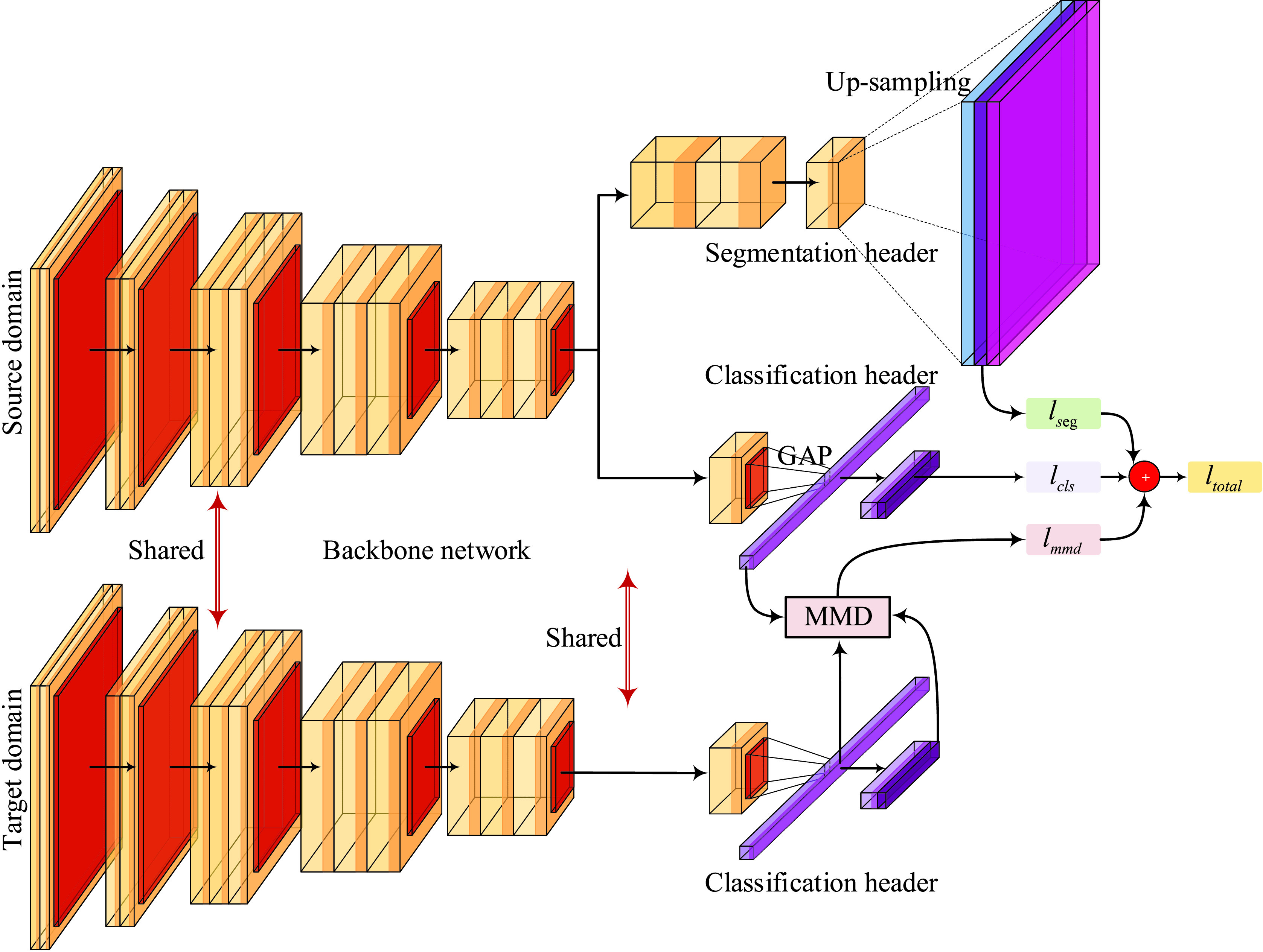
Illustration of the framework of our FCN-based network equipped with a MMD-based domain adaptation module. GAP denotes global average pooling.

We design a FCN-based network as depicted in Fig. 3. It consists of a backbone network, a classification header and a segmentation header. Compared with FCN [16], our model has an auxiliary classification header. The classification header is designed for two purposes. One is to enable our model to perform both classification task and segmentation task for automated infection measurement and COVID-19 diagnosis. The other one is to facilitate the use of MMD-based methods for domain adaptation. The backbone network is responsible for extracting deep features by performing the convolution and spatial pooling operations on DRRs and CXRs. The extracted deep features are then fed into the classification header and segmentation header separately. In the classification branch, we adopt a very simple structure with a global average pooling (GAP) layer and a fully convolution (FC) layer. In the segmentation branch, we use two convolutional layers followed by an up-sampling layer to generate the segmentation output with the same size as the input DRRs and CXRs.

#### 2) MMD-Based Domain Adaptation Module

As a nonparametric distance estimate between two distributions, MMD [45] has been widely used in domain adaptation algorithms to measure the discrepancy between the source and target distributions. In our implementation, we adopt an off-the-shelf MMD-based domain adaptation approach, i.e., LMMD loss proposed by Zhu *et al.* [31]. LMMD can measure the discrepancy of local distributions by taking the correlations of the relevant subdomains into consideration. By minimizing the LMMD loss during the training of deep models, the distributions of relevant subdomains within the same category in the source domain and target domain are drawn close. As the LMMD method is proposed in the context of object recognition and digit classification tasks, we apply it to the classification header directly by aligning the deep features from the GAP layer. The effect of feature alignment can be propagated to the segmentation branch implicitly through the input of the GAP layer. The experiment results have verified the efficacy of our design, which will be detailed in Section IV-D.

#### 3) Objective Function

The training of our model is performed by minimizing the classification loss $l_{cls}$, segmentation loss $l_{seg}$, and LMMD loss $l_{mmd}$ simultaneously. The total loss is computed as

\begin{equation*} l_{total}=\lambda _{cls}\times l_{cls}+\lambda _{seg}\times l_{seg}+\lambda _{mmd}\times l_{mmd}\tag{5}\end{equation*}

where $\lambda _{cls}$, $\lambda _{seg}$, and $\lambda _{mmd}$ denote the weights of the classification loss, segmentation loss, and LMMD loss, respectively.

## IV. Experiments and Results

### A. Materials

#### 1) Chest CT Scans

We use the public COVID-19-CT-Seg dataset [55], which consists of 20 public COVID-19 CT cases with voxel-level annotations of the left lung, right lung and COVID-19 infection. The annotations, first labeled by junior annotators, are refined by two radiologists with 5 years of experience, and are further verified and refined by a senior radiologist with more than 10 years of experience in chest radiology. In these 20 CT volumes, the voxel values of 10 volumes have been normalized to [0, 255], and thus we cannot access their CT values measured in HUs. We discard these ten cases and use the other 10 CT cases for the synthesis of DRRs in our experiments. For each CT case, we obtain 40 front-view DRRs and 40 lateral-view DRRs with pixel-level annotations of infected regions by using our infection-aware DRR generator, which will be detailed in Section IV-B. Thus, we build a training set in the source domain with these 800 DRRs as shown in Table 1.

**TABLE 1:** The Split of Training, Validation and Test Sets

Source domain (DRRs)	Target domain (CXRs)	
Training set	Training-validation set	Test set
800 DRRs with pixel-level annotations of COVID-19 infection	219 positive images	558 positive images
	219 negative images	558 negative images

#### 2) Chest X-Ray Images

We use two public COVID-19 CXR collections [56], [57], which are constructed upon Radiopaedia [58], COVID-19 image data collection [59], Chest X-Ray Images (Pneumonia) [60], SIRM [61], Twitter COVID-19 CXR dataset [62], and Hannover Medical School dataset [63]. The first collection [56] consists of 219 COVID-19 positive images from 96 patients and 1341 normal images from 1211 patients. The second collection contains 558 COVID-19 positive images that are different from the 219 positive images in the first collection. We randomly select 219 normal images from 219 different patients in the first collection, and combine them with the 219 COVID-19 positive images in the first collection to build a training-validation set in the target domain. Besides, we use the 558 COVID-19 positive images in the second collection and 558 normal images that are randomly selected from the remaining 992 patients in the first collection to build an independent test set in the target domain as shown in Table 1. We perform 4-fold cross validation (75% patients for training and 25% patients for testing) on the training-validation set and perform independent testing on the test set. Note that these CXRs have no pixel-level expert annotations of infected regions. We can only use the image tags (i.e., positive or negative) to evaluate the classification and segmentation results. Therefore, we also introduce another CXR dataset, i.e., the BIMCV COVID-19+ dataset [64], where a sub-set of 10 CXRs is annotated with ROIs of the COVID-19 findings (e.g., consolidation/GGOs) by a team of eight radiologists from the Hospital Universitario de San Juan de Alicante for the first iteration. To our best knowledge, this is the only COVID-19 CXR dataset that provides pixel-level annotations of infected regions currently. Although the sub-set is very small, we use it to provide preliminary insights into the real infection segmentation performance of our method.

### B. Infection-Aware DRRs

#### 1) Generating Normal DRRs

**FIGURE 4.**
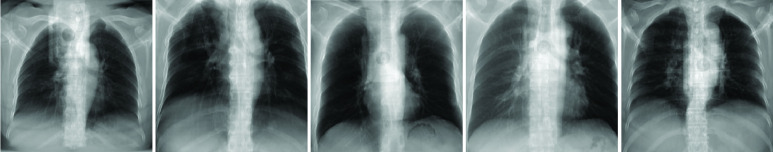
Illustration of normal DRRs generated by our infection-aware DRR generator.

DRRs with no findings are important for training deep classification and segmentation models for COVID-19 diagnosis. Our infection-aware generator is able to generate such DRRs with no findings by setting the weight of infected voxels to a relatively small value to reduce the CRIV in the ray-casting process. In our experiment, we empirically set the weights of background, lung, and COVID-19 infection as $w_{0}=24.0$, $w_{1}=24.0$, and $w_{2}=1.0$. Several synthetic normal DRRs are depicted in Fig. 4.

#### 2) Generating Multiple DRRs From a Single CT Volume

**FIGURE 5.**
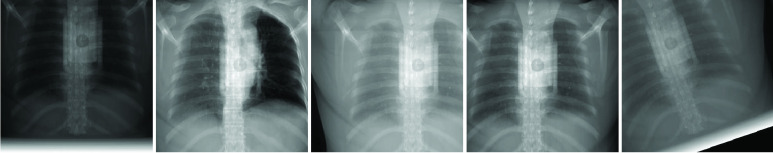
Illustration of multiple DRRs from a single CT volume by adjusting the pose of this CT volume.

It is easy to generate multiple DRRs from a single CT volume by adjusting the pose (position and orientation) of the CT volume within a virtual imaging system. In our experiment, we randomly shift each CT volume between −100 and 100, and rotate it between − 45° and 45° in 3D directions. Several DRRs generated from a single CT volume are illustrated in Fig. 5.

#### 3) Generating DRRs With Different Strengths of Radiological Signs of COVID-19 Infection

**FIGURE 6.**
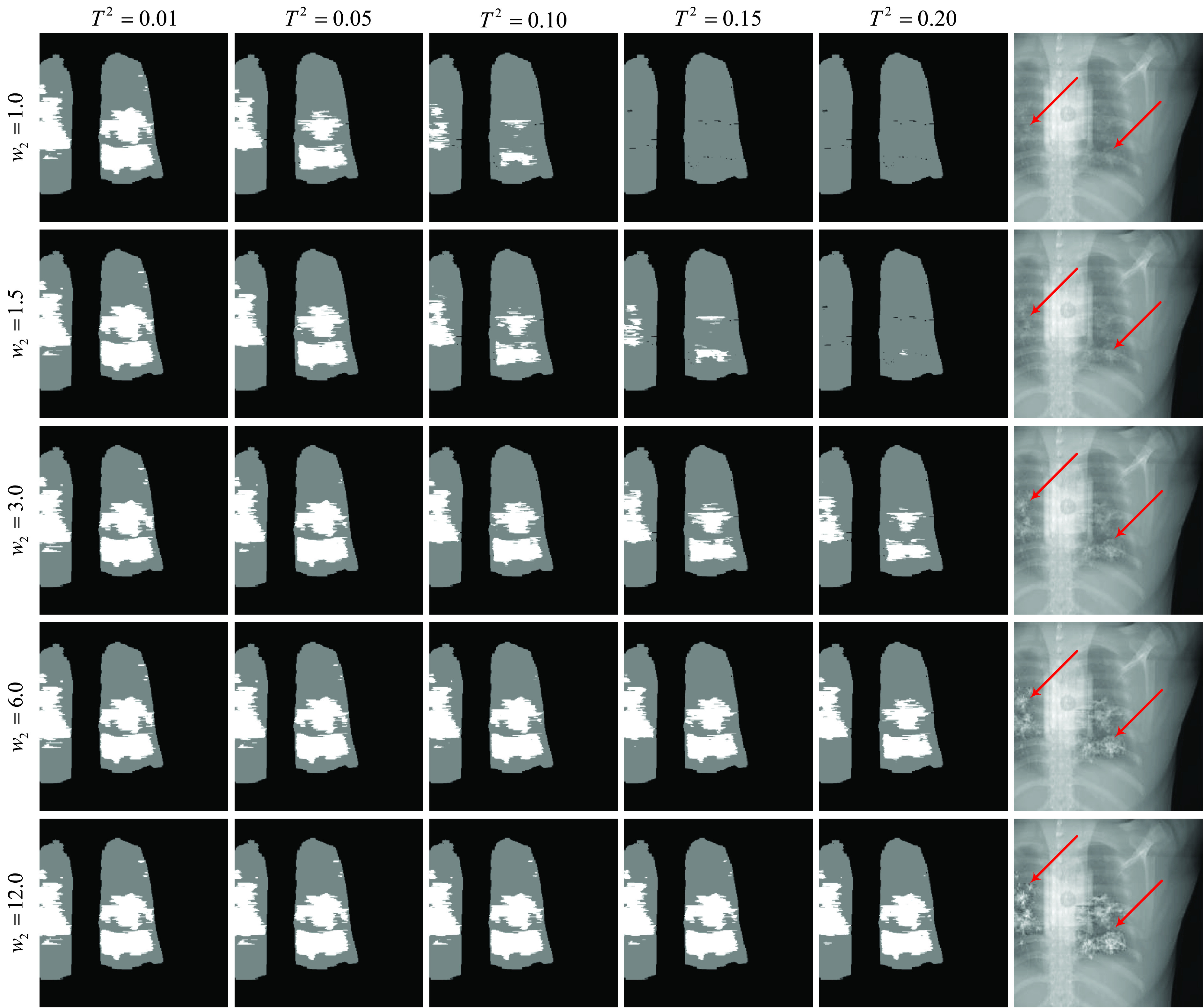
Illustration of DRRs with different strengths of radiological signs of COVID-19 infection from a single CT volume and their corresponding pixel-level annotations of the lungs and infected regions. The weights of background and lungs are set as 1.0, and the contribution threshold of the lungs $T^{1}$ is set as 0.0. The red arrows in the last column highlight the infected regions.

Our infection-aware DRR generator is able to generate DRRs with different strengths of radiological signs of COVID-19 infection by adjusting the weights of background, lung and COVID-19 infection $(w_{0},w_{1},w_{2})$. In our experiment, we set $(w_{0},w_{1},w_{2})$ to (12.0, 12.0, 1.0), (6.0, 6.0, 1.0), (3.0, 3.0, 1.0), (1.5, 1.5, 1.0), (1.0, 1.0, 1.0), (1.0, 1.0, 1.5), (1.0, 1.0, 3.0), (1.0, 1.0, 6.0), and (1.0, 1.0, 12.0) separately. Several samples are shown in the last column of Fig. 6.

#### 4) Generating Pixel-Level Annotations of COVID-19 Infection

We empirically set the contribution threshold of infected voxels (CTIV) $T^{2}$ as 0.00, 0.01, 0.05, 0.10, 0.15, 0.20, and 0.40 respectively to get the corresponding infection annotation masks. The contribution threshold of the lungs is set to 0.00. Several infection masks are visualized in the first five columns of Fig. 6.

#### 5) Building Training Sets in the Source Domain (DRRs)

For each CT volume, we first generate 40 normal DRRs, including 20 front-view DRRs and 20 lateral-view DRRs by randomly adjusting its pose. Next, in the same way we generate 40 DRRs and the corresponding pixel-level annotations of infected regions with given $(w_{0},w_{1},w_{2})$ and $T^{2}$. By this means, we build a training set in the source domain with 800 DRRs as shown in Table 1. Finally, with given the 63 different combinations of $(w_{0},w_{1},w_{2})$ and $T^{2}$, we totally obtain 63 training sets in the source domain.

### C. Experiment Setting

#### 1) Experiment Design

This article aims at learning automated COVID-19 infection segmentation on CXRs from DRRs. To this end, we propose DRR4Covid, which consists of an infection-aware DRR generator, a FCN-based network and a MMD-based domain adaptation module. To verify the efficacy of our method, we conduct experiments from four aspects: 1) standard DRRs versus infection-aware DRRs; 2) using domain adaptation versus not using domain adaptation; 3) estimating the detection limit of X-ray imaging in detecting COVID-19 infection by searching for the best parameters $(w_{0},w_{1},w_{2})$ and $T^{2}$; and 4) evaluation of segmentation performance on 10 CXRs. Accordingly, in each fold, we first train the FCN-based network on the 63 training sets in the source domain without using the domain adaptation module respectively. Next, we train the same network on the 63 training sets in the source domain and the training set in the target domain by using the MMD-based domain adaptation module separately. All of the trained models are finally evaluated on the same validation set and test set. We report the results of the 4-fold cross validation in the format of Mean ± Standard Deviation. Note that the annotations of CXRs in the target domain are always kept unseen in all training tasks and our infection-aware DRR generator will produce standard DRRs when $(w_{0},w_{1},w_{2})$ equals to (1.0, 1.0, 1.0).

#### 2) Training Details

ResNet-18 is adopted as the backbone of the FCN-based network in our experiments. We train the network with 100 epochs by using Adam optimizer with the parameters of $\beta _{1}=0.9$ and $\beta _{2}=0.999$. We adopt mini-batch of 16, and use an initial learning rate of 0.0001 that is linearly decayed by 2% each epoch after 50 epochs. We initialize the backbone network with the weights of ResNet-18 that are pre-trained on ImageNet. Data augmentation, involving random cropping, horizontal flipping, vertical flipping and random rotating, are performed. The input image size of our network is $256\times 256\times 3$. Besides, the category-weighted cross entropy loss is adopted as the segmentation loss to emphasize the optimization of COVID-19 infection segmentation, where the weights of background, lung and COVID-19 infection are set to 0.1, 1.0 and 5.0. Binary cross entropy loss is used as the classification loss. The weights of the classification loss, segmentation loss and LMMD loss are set as $\lambda _{cls}=1.0$, $\lambda _{seg}=1.0$, and $\lambda _{mmd}=0.3$ respectively. We use the PyTorch1.4 framework to build the deep models. The infection-aware DRR generator is designed by using CUDA10.2, Python3.6, and Cython. All models are trained and evaluated on a Linux server equipped with four NVIDIA GTX1080ti GPU cards.

#### 3) Evaluation Metrics

For the classification output of our model, we adopt the commonly used classification metrics, including accuracy, F1-score and area under precision-recall curve (AUC of PR-curve). As the pixel-level annotations of infected regions are not available for the validation and test sets in the target domain (CXRs), we are unable to use the segmentation evaluation metrics directly. To enable evaluate the quality of segmentation output of our model, we convert the segmentation output into classification output by determining whether there exists infected regions in the segmentation output, and then adopt the same three classification metrics. As for the sub-set of 10 CXRs in the BIMCV COVID-19+ dataset, we directly adopt the commonly used segmentation metric, i.e., Dice similarity coefficient (DSC), to evaluate the segmentation results.

#### 4) Statistical Analysis

Statistical tests are conducted to determine the significance in performance differences of learning infection segmentation from DRRs between standard DRRs and our infection-aware DRRs and between using our domain adaptation module and not using domain adaptation. According to the experiment design, we perform paired samples t-test to compare the means of scores. Specifically, we remove the outliers of the scores and apply Shaprio-Wilk test for normality. If the variables violate the assumption of normality, we perform Wilcoxon signed-rank test instead of paired samples t-test. The SciPy package is used in these analyses.

### D. Experiment Results

We report the evaluation results of our model trained on the 63 training sets with/without MMD-based domain adaptation module in Table 4–27 of the appendix. We will analyze these results from the four perspectives as introduced in the experiment design.

**TABLE 2:** Total Average (T-Avg.) Scores (Mean ± Standard Deviation) of Our Model Trained on 63 Different Training Sets in the Source Domain. Each Item in This Table is the Average of the Corresponding Items in Table 4–27 of the Appendix. EAPIV Denotes the Equivalent Average Percent Infection Volume of the Lungs of the 10 CT Cases That are Used for the Synthesis of Infection-Aware DRRs

T-Avg(%)	$w_{0,1}=12.0$	$w_{0,1}=6.0$	$w_{0,1}=3.0$	$w_{0,1}=1.5$	$w_{0,1}=1.0$	$w_{0,1}=1.0$	$w_{0,1}=1.0$	$w_{0,1}=1.0$	$w_{0,1}=1.0$
	$w_{2}=1.0$	$w_{2}=1.0$	$w_{2}=1.0$	$w_{2}=1.0$	$w_{2}=1.0$	$w_{2}=1.5$	$w_{2}=3.0$	$w_{2}=6.0$	$w_{2}=12.0$
$T^{2}=0.40$	32.1±24.5	31.3±24.1	31.2±24.6	36.4±27.9	42.7±32.4	55.4±30.1	81.0±13.6	82.5±14.1	76.2±19.1
$T^{2}=0.20$	30.9±24.2	30.3±23.6	40.8±29.8	34.7±20.3	50.2±11.5	75.4±18.2	87.9±11.4	85.2±13.3	75.8±19.5
$T^{2}=0.15$	32.7±25.0	36.3±28.4	27.8±21.7	35.7±11.7	45.7±10.6	76.8±16.3	87.0±12.5	83.8±12.9	79.5±20.1
$T^{2}=0.10$	28.6±22.9	38.2±30.0	31.5±19.2	32.9±12.2	54.9±16.2	77.3±14.7	86.8±12.7	83.7±13.5	75.2±19.6
$T^{2}=0.05$	38.7±29.6	39.7±22.2	28.3±10.5	36.3±12.7	53.8±9.8	80.4±15.4	86.8±11.9	83.4±12.1	78.7±17.9
$T^{2}=0.01$	40.1±18.2	38.6±15.7	31.7±12.9	36.9±14.0	54.9±13.9	78.5±14.9	86.4±12.4	83.0±13.9	75.0±19.4
$T^{2}=0.00$	46.4±15.4	40.7±15.3	30.9±12.9	39.4±13.5	55.9±11.9	79.7±16.1	86.9±12.7	85.0±13.1	73.8±18.3
EAPIV (%)	0.85±0.98	1.67±1.90	3.22±3.57	6.03±6.37	8.51±8.63	11.77±11.30	19.43±16.29	29.75±20.56	42.30±22.57

**TABLE 3:** DSC (Mean ± Standard Deviation) Table of Segmentation Results on the Sub-Set of 10 CXRs

DSC(%)	$w_{0,1}=12.0$	$w_{0,1}=6.0$	$w_{0,1}=3.0$	$w_{0,1}=1.5$	$w_{0,1}=1.0$	$w_{0,1}=1.0$	$w_{0,1}=1.0$	$w_{0,1}=1.0$	$w_{0,1}=1.0$
	$w_{2}=1.0$	$w_{2}=1.0$	$w_{2}=1.0$	$w_{2}=1.0$	$w_{2}=1.0$	$w_{2}=1.5$	$w_{2}=3.0$	$w_{2}=6.0$	$w_{2}=12.0$
$T^{2}=0.40$	0.0±0.0	0.0±0.0	0.0±0.0	0.0±0.0	0.0±0.0	0.0±0.0	5.7±8.9	28.6±15.2	33.7±14.1
$T^{2}=0.20$	0.0±0.0	0.0±0.0	0.0±0.0	0.0±0.0	0.0±0.0	8.3±10.7	37.3±14.2	37.9±14.8	39.3±16.6
$T^{2}=0.15$	0.0±0.0	0.0±0.0	0.0±0.0	0.0±0.0	0.5±1.6	21.4±15.3	39.6±15.3	42.1±14.5	39.3±13.9
$T^{2}=0.10$	0.0±0.0	0.0±0.0	0.0±0.0	0.0±0.0	4.1±8.3	37.0±13.6	40.9±14.6	40.1±16.1	36.3±17.8
$T^{2}=0.05$	0.0±0.0	0.0±0.0	0.0±0.0	0.0±0.0	7.5±11.2	41.7±14.9	42.4±15.4	41.8±15.1	37.8±16.5
$T^{2}=0.01$	5.7±11.8	3.8±12.2	0.1±0.3	0.5±2.7	12.1±16.1	39.5±16.0	42.8±15.3	41.5±15.2	37.2±17.5
$T^{2}=0.00$	26.2±16.9	6.8±10.4	0.0±0.0	1.5±5.4	11.4±15.9	39.7±16.9	43.6±16.0	41.1±15.3	30.9±20.1

**TABLE 4:** Accuracy (Mean ± Standard Deviation) Table of Classification Output on Validation Set in the Target Domain (Domain Adaptation)

Accuracy (%)	$w_{0,1}=12.0$	$w_{0,1}=6.0$	$w_{0,1}=3.0$	$w_{0,1}=1.5$	$w_{0,1}=1.0$	$w_{0,1}=1.0$	$w_{0,1}=1.0$	$w_{0,1}=1.0$	$w_{0,1}=1.0$
	$w_{2}=1.0$	$w_{2}=1.0$	$w_{2}=1.0$	$w_{2}=1.0$	$w_{2}=1.0$	$w_{2}=1.5$	$w_{2}=3.0$	$w_{2}=6.0$	$w_{2}=12.0$
$T^{2}=0.40$	50.0±0.0	50.0±0.0	50.0±0.0	50.0±0.0	51.0±0.7	68.7±6.3	93.1±2.6	89.9±6.2	89.4±3.2
$T^{2}=0.20$	50.0±0.0	50.0±0.0	50.8±1.3	16.6±3.9	40.6±6.0	90.7±4.1	90.9±4.6	90.8±5.6	86.3±4.0
$T^{2}=0.15$	50.0±0.0	50.0±0.0	23.4±14.9	23.9±1.3	40.6±1.9	84.9±13.1	89.2±4.4	90.1±5.2	91.6±1.8
$T^{2}=0.10$	50.0±0.0	50.0±0.0	17.6±3.5	21.8±2.7	53.5±21.1	88.6±6.3	90.9±4.2	90.5±2.3	84.8±4.4
$T^{2}=0.05$	50.0±0.0	47.0±13.0	21.3±3.6	29.8±6.1	51.1±2.9	93.0±1.3	90.6±5.7	87.2±3.8	87.4±3.1
$T^{2}=0.01$	45.1±3.7	23.6±5.0	26.4±3.5	33.1±8.2	57.4±10.9	85.4±13.0	90.2±5.4	90.2±2.7	82.3±8.1
$T^{2}=0.00$	52.3±5.2	29.1±2.4	24.9±3.5	38.1±4.6	57.0±7.6	92.7±2.9	91.1±3.0	92.9±0.6	82.1±10.5

**TABLE 5:** AUC (Mean ± Standard Deviation) Table of Classification Output on Validation Set in the Target Domain (Domain Adaptation)

AUC (%)	$w_{0,1}=12.0$	$w_{0,1}=6.0$	$w_{0,1}=3.0$	$w_{0,1}=1.5$	$w_{0,1}=1.0$	$w_{0,1}=1.0$	$w_{0,1}=1.0$	$w_{0,1}=1.0$	$w_{0,1}=1.0$
	$w_{2}=1.0$	$w_{2}=1.0$	$w_{2}=1.0$	$w_{2}=1.0$	$w_{2}=1.0$	$w_{2}=1.5$	$w_{2}=3.0$	$w_{2}=6.0$	$w_{2}=12.0$
$T^{2}=0.40$	55.8±7.3	57.0±4.4	56.4±15.6	57.2±14.9	87.4±11.2	95.2±2.8	98.6±0.8	95.1±4.0	95.2±2.3
$T^{2}=0.20$	51.5±15.6	46.2±6.0	64.3±15.4	6.3±2.9	34.7±10.6	95.6±1.0	96.9±1.9	98.1±0.9	92.5±6.1
$T^{2}=0.15$	50.9±6.9	50.6±16.0	9.7±2.3	11.6±3.6	31.1±10.0	90.0±13.9	98.2±0.8	94.2±5.9	97.3±0.9
$T^{2}=0.10$	45.4±8.1	79.3±6.0	8.2±3.0	10.4±4.4	49.0±29.2	93.9±4.5	97.4±0.9	96.0±1.5	90.3±7.2
$T^{2}=0.05$	75.2±17.3	40.1±26.4	8.4±4.2	16.3±9.7	48.7±8.1	98.0±0.5	98.4±0.6	94.2±4.0	92.5±1.4
$T^{2}=0.01$	21.0±10.6	10.9±3.7	9.4±4.2	16.7±8.4	57.8±21.1	90.4±11.8	98.8±0.7	97.0±0.9	86.9±10.9
$T^{2}=0.00$	47.6±13.6	11.1±3.4	8.9±4.0	22.5±6.2	60.4±15.7	98.0±1.0	98.4±0.6	97.6±1.2	81.3±20.0

**TABLE 6:** F1-Score (Mean ± Standard Deviation) Table of Classification Output on Validation Set in the Target Domain (Domain Adaptation)

F1-score (%)	$w_{0,1}=12.0$	$w_{0,1}=6.0$	$w_{0,1}=3.0$	$w_{0,1}=1.5$	$w_{0,1}=1.0$	$w_{0,1}=1.0$	$w_{0,1}=1.0$	$w_{0,1}=1.0$	$w_{0,1}=1.0$
	$w_{2}=1.0$	$w_{2}=1.0$	$w_{2}=1.0$	$w_{2}=1.0$	$w_{2}=1.0$	$w_{2}=1.5$	$w_{2}=3.0$	$w_{2}=6.0$	$w_{2}=12.0$
$T^{2}=0.40$	0.0±0.0	0.0±0.0	0.0±0.0	0.0±0.0	3.7±2.7	53.3±13.5	92.6±2.9	88.2±8.2	88.0±4.1
$T^{2}=0.20$	0.0±0.0	0.0±0.0	2.9±5.0	26.2±7.2	55.1±5.4	90.7±3.9	89.7±5.8	89.6±6.8	84.0±5.4
$T^{2}=0.15$	0.0±0.0	0.0±0.0	17.1±8.8	37.9±1.7	57.4±2.0	86.7±9.2	87.7±5.9	88.7±6.6	90.8±2.2
$T^{2}=0.10$	0.0±0.0	0.0±0.0	16.3±5.8	35.2±3.9	64.1±14.5	89.2±5.2	89.9±5.4	89.5±2.9	81.8±6.0
$T^{2}=0.05$	0.0±0.0	13.4±13.6	32.9±5.5	45.0±7.7	62.2±1.6	92.7±1.5	89.2±7.3	85.1±5.1	85.5±4.1
$T^{2}=0.01$	5.7±3.1	35.5±6.3	39.6±4.1	47.6±9.4	65.5±6.0	86.2±10.6	88.8±7.0	89.1±3.4	77.4±13.7
$T^{2}=0.00$	34.0±2.9	42.0±3.6	37.5±4.3	53.3±6.1	65.1±3.4	92.3±3.2	90.2±3.7	92.3±0.6	76.0±17.1

**TABLE 7:** Accuracy (Mean ± Standard Deviation) Table of Segmentation Output on Validation Set in the Target Domain (Domain Adaptation)

Accuracy (%)	$w_{0,1}=12.0$	$w_{0,1}=6.0$	$w_{0,1}=3.0$	$w_{0,1}=1.5$	$w_{0,1}=1.0$	$w_{0,1}=1.0$	$w_{0,1}=1.0$	$w_{0,1}=1.0$	$w_{0,1}=1.0$
	$w_{2}=1.0$	$w_{2}=1.0$	$w_{2}=1.0$	$w_{2}=1.0$	$w_{2}=1.0$	$w_{2}=1.5$	$w_{2}=3.0$	$w_{2}=6.0$	$w_{2}=12.0$
$T^{2}=0.40$	50.0±0.0	50.0±0.0	50.0±0.0	50.0±0.0	50.4±0.7	52.8±3.0	75.5±2.1	92.1±6.1	90.5±2.2
$T^{2}=0.20$	50.0±0.0	50.0±0.0	50.4±0.5	46.3±2.4	48.3±11.5	83.5±6.6	91.9±3.8	92.9±3.3	89.8±5.4
$T^{2}=0.15$	50.0±0.0	50.0±0.0	49.8±0.8	38.8±7.1	37.3±3.0	81.4±14.2	91.2±4.5	92.8±4.4	94.2±1.7
$T^{2}=0.10$	50.0±0.0	50.0±0.0	34.1±7.7	21.9±2.3	48.4±14.0	79.7±5.5	90.9±2.6	92.8±1.4	88.3±4.2
$T^{2}=0.05$	50.0±0.0	45.5±6.2	20.2±2.3	33.3±6.4	46.9±2.6	85.0±6.3	91.4±3.9	89.9±3.3	90.4±4.1
$T^{2}=0.01$	40.1±3.2	40.5±2.1	32.5±3.4	37.0±6.4	50.6±9.4	82.2±15.1	90.3±3.6	91.5±2.6	86.5±8.8
$T^{2}=0.00$	48.4±1.7	43.3±3.1	33.7±2.9	39.7±4.0	48.1±3.0	88.1±1.9	92.5±2.9	95.3±1.4	85.4±9.6

**TABLE 8:** AUC (Mean ± Standard Deviation) Table of Segmentation Output on Validation Set in the Target Domain (Domain Adaptation)

AUC (%)	$w_{0,1}=12.0$	$w_{0,1}=6.0$	$w_{0,1}=3.0$	$w_{0,1}=1.5$	$w_{0,1}=1.0$	$w_{0,1}=1.0$	$w_{0,1}=1.0$	$w_{0,1}=1.0$	$w_{0,1}=1.0$
	$w_{2}=1.0$	$w_{2}=1.0$	$w_{2}=1.0$	$w_{2}=1.0$	$w_{2}=1.0$	$w_{2}=1.5$	$w_{2}=3.0$	$w_{2}=6.0$	$w_{2}=12.0$
$T^{2}=0.40$	54.0±15.2	38.7±17.5	55.9±15.2	60.2±17.6	82.9±7.8	89.1±3.9	94.7±0.8	97.3±1.3	92.5±3.9
$T^{2}=0.20$	52.3±16.4	41.0±20.5	78.9±6.5	15.3±4.9	44.0±13.4	89.9±5.8	97.7±1.8	97.7±1.6	90.8±7.2
$T^{2}=0.15$	51.8±21.1	55.8±16.9	25.3±9.9	27.6±6.5	34.5±2.4	89.1±9.0	96.3±1.6	93.0±7.4	94.1±3.3
$T^{2}=0.10$	46.9±15.1	65.7±6.8	14.2±5.6	17.4±5.6	48.4±23.8	92.3±4.6	95.2±2.8	93.9±3.4	88.0±8.6
$T^{2}=0.05$	52.6±14.7	34.5±18.5	12.7±3.1	19.4±5.6	51.6±3.7	95.5±1.1	90.0±8.2	92.9±6.9	87.2±5.4
$T^{2}=0.01$	27.8±2.8	11.8±3.5	9.6±4.6	16.1±7.6	52.5±16.4	87.9±11.8	92.0±5.1	93.0±5.2	85.7±11.6
$T^{2}=0.00$	33.3±2.8	10.4±3.1	9.4±3.7	17.9±2.8	52.2±10.4	94.0±3.0	92.9±5.5	97.1±1.2	86.1±9.1

**TABLE 9:** F1-Score (Mean ± Standard Deviation) Table of Segmentation Output on Validation Set in the Target Domain (Domain Adaptation)

F1-score (%)	$w_{0,1}=12.0$	$w_{0,1}=6.0$	$w_{0,1}=3.0$	$w_{0,1}=1.5$	$w_{0,1}=1.0$	$w_{0,1}=1.0$	$w_{0,1}=1.0$	$w_{0,1}=1.0$	$w_{0,1}=1.0$
	$w_{2}=1.0$	$w_{2}=1.0$	$w_{2}=1.0$	$w_{2}=1.0$	$w_{2}=1.0$	$w_{2}=1.5$	$w_{2}=3.0$	$w_{2}=6.0$	$w_{2}=12.0$
$T^{2}=0.40$	0.0±0.0	0.0±0.0	0.0±0.0	0.0±0.0	1.6±2.7	10.8±9.9	67.4±3.7	90.9±7.9	89.5±2.7
$T^{2}=0.20$	0.0±0.0	0.0±0.0	1.8±1.8	8.4±1.5	45.1±10.7	83.1±6.3	91.0±4.7	92.2±3.9	88.2±6.7
$T^{2}=0.15$	0.0±0.0	0.0±0.0	1.0±1.7	30.6±7.7	52.5±3.8	84.2±8.9	90.2±5.8	92.1±5.4	93.8±1.8
$T^{2}=0.10$	0.0±0.0	0.0±0.0	7.6±2.9	33.4±4.2	61.5±9.4	82.5±3.5	90.1±3.4	92.3±1.7	86.5±5.4
$T^{2}=0.05$	0.0±0.0	8.0±3.3	31.4±3.9	49.3±7.2	61.5±4.1	86.6±4.8	90.5±4.9	88.7±4.2	89.2±5.0
$T^{2}=0.01$	57.2±3.3	57.5±2.4	48.1±3.9	53.2±6.8	63.3±5.9	84.6±11.2	89.3±4.7	90.8±3.2	83.1±13.5
$T^{2}=0.00$	65.3±1.5	60.2±3.1	49.3±3.6	56.1±4.3	61.6±3.0	88.8±1.6	92.0±3.3	95.0±1.6	81.3±14.5

**TABLE 10:** Accuracy (Mean ± Standard Deviation) Table of Classification Output on Test Set in the Target Domain (Domain Adaptation)

Accuracy (%)	$w_{0,1}=12.0$	$w_{0,1}=6.0$	$w_{0,1}=3.0$	$w_{0,1}=1.5$	$w_{0,1}=1.0$	$w_{0,1}=1.0$	$w_{0,1}=1.0$	$w_{0,1}=1.0$	$w_{0,1}=1.0$
	$w_{2}=1.0$	$w_{2}=1.0$	$w_{2}=1.0$	$w_{2}=1.0$	$w_{2}=1.0$	$w_{2}=1.5$	$w_{2}=3.0$	$w_{2}=6.0$	$w_{2}=12.0$
$T^{2}=0.40$	50.0±0.0	50.0±0.0	50.0±0.0	50.0±0.1	51.2±0.7	75.5±6.0	95.1±0.7	93.3±2.2	93.0±2.1
$T^{2}=0.20$	50.0±0.0	50.0±0.0	50.4±0.4	20.8±5.4	47.9±4.9	92.8±2.8	94.9±0.9	95.3±0.7	91.1±2.4
$T^{2}=0.15$	50.0±0.0	50.0±0.0	25.4±12.0	30.4±0.6	46.4±1.7	87.2±12.8	94.6±0.7	93.6±3.2	93.8±1.5
$T^{2}=0.10$	50.0±0.0	50.0±0.0	21.0±5.0	27.3±2.3	59.7±19.9	90.0±4.6	94.5±0.4	93.9±1.1	89.2±4.6
$T^{2}=0.05$	50.0±0.0	46.7±11.0	27.0±3.2	35.2±5.4	58.4±4.7	94.2±1.7	94.1±1.0	92.1±1.5	91.7±1.1
$T^{2}=0.01$	44.9±4.5	25.4±6.6	32.5±2.3	37.6±7.5	63.8±11.6	88.6±10.7	95.0±0.4	94.2±1.0	88.3±5.9
$T^{2}=0.00$	48.3±5.6	34.0±1.5	30.7±2.3	44.5±5.0	64.3±10.3	94.9±0.6	95.4±0.5	94.6±0.8	87.5±8.5

**TABLE 11:** AUC (Mean ± Standard Deviation) Table of Classification Output on Test Set in the Target Domain (Domain Adaptation)

AUC (%)	$w_{0,1}=12.0$	$w_{0,1}=6.0$	$w_{0,1}=3.0$	$w_{0,1}=1.5$	$w_{0,1}=1.0$	$w_{0,1}=1.0$	$w_{0,1}=1.0$	$w_{0,1}=1.0$	$w_{0,1}=1.0$
	$w_{2}=1.0$	$w_{2}=1.0$	$w_{2}=1.0$	$w_{2}=1.0$	$w_{2}=1.0$	$w_{2}=1.5$	$w_{2}=3.0$	$w_{2}=6.0$	$w_{2}=12.0$
$T^{2}=0.40$	57.8±10.8	52.6±9.3	50.8±13.6	57.2±11.3	85.6±10.5	98.5±0.6	98.1±0.3	97.6±1.7	96.7±1.1
$T^{2}=0.20$	47.0±9.7	50.4±12.5	66.5±13.2	11.6±4.9	45.6±6.7	97.2±1.3	98.7±0.5	98.8±0.7	95.2±2.2
$T^{2}=0.15$	48.1±11.3	56.6±16.8	11.8±2.5	17.2±3.9	35.0±6.4	90.2±13.8	98.9±0.8	97.5±2.0	97.7±0.5
$T^{2}=0.10$	41.8±16.3	81.8±7.0	11.8±5.5	14.4±1.1	55.1±24.3	95.3±2.9	98.5±0.7	98.4±0.3	92.6±7.0
$T^{2}=0.05$	80.9±10.9	42.5±24.2	12.1±2.0	20.1±3.6	54.2±15.6	98.1±0.5	99.0±0.6	97.7±0.7	96.3±0.6
$T^{2}=0.01$	17.1±7.9	13.2±2.6	14.2±2.9	20.7±6.6	64.9±20.3	92.2±11.0	98.9±0.7	98.6±0.4	92.5±5.1
$T^{2}=0.00$	38.2±11.5	15.9±2.9	12.5±2.6	27.4±6.3	68.9±16.0	98.5±0.5	99.1±0.2	98.9±0.2	89.6±11.7

**TABLE 12:** F1-Score (Mean ± Standard Deviation) Table of Classification Output on Test Set in the Target Domain (Domain Adaptation)

F1-score (%)	$w_{0,1}=12.0$	$w_{0,1}=6.0$	$w_{0,1}=3.0$	$w_{0,1}=1.5$	$w_{0,1}=1.0$	$w_{0,1}=1.0$	$w_{0,1}=1.0$	$w_{0,1}=1.0$	$w_{0,1}=1.0$
	$w_{2}=1.0$	$w_{2}=1.0$	$w_{2}=1.0$	$w_{2}=1.0$	$w_{2}=1.0$	$w_{2}=1.5$	$w_{2}=3.0$	$w_{2}=6.0$	$w_{2}=12.0$
$T^{2}=0.40$	0.0±0.0	0.0±0.0	0.0±0.0	0.2±0.3	4.9±2.7	66.7±10.6	95.0±0.6	92.8±2.6	92.5±2.4
$T^{2}=0.20$	0.0±0.0	0.0±0.0	1.5±1.6	28.7±7.2	61.9±2.8	92.9±2.6	94.7±1.0	95.2±0.8	90.2±2.9
$T^{2}=0.15$	0.0±0.0	0.0±0.0	19.0±9.4	44.0±1.8	62.8±1.6	89.2±9.2	94.4±0.9	93.1±3.8	93.5±1.6
$T^{2}=0.10$	0.0±0.0	0.0±0.0	16.3±6.8	41.0±4.0	70.6±13.4	90.7±3.7	94.5±0.4	93.5±1.3	87.6±6.0
$T^{2}=0.05$	0.0±0.0	9.5±9.7	39.0±4.8	50.9±6.6	68.9±2.5	94.2±1.5	93.9±1.2	91.5±1.8	91.0±1.3
$T^{2}=0.01$	4.5±3.6	35.1±9.7	46.0±2.9	52.5±9.0	72.4±6.4	89.8±8.3	94.9±0.4	93.9±1.0	86.5±8.2
$T^{2}=0.00$	24.3±5.2	46.8±2.0	44.4±1.9	59.8±5.1	72.3±5.8	94.8±0.6	95.3±0.5	94.4±0.8	84.5±12.1

**TABLE 13:** Accuracy (Mean ± Standard Deviation) Table of Segmentation Output on Test Set in the Target Domain (Domain Adaptation)

Accuracy (%)	$w_{0,1}=12.0$	$w_{0,1}=6.0$	$w_{0,1}=3.0$	$w_{0,1}=1.5$	$w_{0,1}=1.0$	$w_{0,1}=1.0$	$w_{0,1}=1.0$	$w_{0,1}=1.0$	$w_{0,1}=1.0$
	$w_{2}=1.0$	$w_{2}=1.0$	$w_{2}=1.0$	$w_{2}=1.0$	$w_{2}=1.0$	$w_{2}=1.5$	$w_{2}=3.0$	$w_{2}=6.0$	$w_{2}=12.0$
$T^{2}=0.40$	50.0±0.0	50.0±0.0	50.0±0.0	50.0±0.0	50.6±0.5	52.4±2.6	79.6±3.0	93.7±1.7	93.8±1.1
$T^{2}=0.20$	50.0±0.0	50.0±0.0	50.4±0.1	47.7±1.0	51.9±9.1	89.2±3.5	95.6±0.6	95.9±0.5	92.3±2.0
$T^{2}=0.15$	50.0±0.0	50.0±0.0	50.2±0.5	41.2±5.0	43.6±1.8	83.7±14.8	95.0±0.6	94.5±2.2	94.5±1.0
$T^{2}=0.10$	50.0±0.0	50.0±0.0	37.7±8.3	26.1±1.9	56.3±14.3	80.8±6.4	94.0±0.7	95.2±0.4	90.8±4.5
$T^{2}=0.05$	50.0±0.0	46.1±7.6	25.0±2.7	41.0±4.0	53.2±2.5	87.4±5.3	94.1±0.9	94.0±1.3	93.3±0.6
$T^{2}=0.01$	43.8±1.5	46.5±1.2	42.6±0.9	43.3±4.3	58.2±10.3	83.7±12.6	94.2±0.9	94.8±0.9	90.0±5.1
$T^{2}=0.00$	49.8±0.1	47.8±1.0	40.2±1.6	46.6±2.6	55.7±4.4	89.2±2.2	94.8±0.6	95.2±0.6	89.3±7.0

**TABLE 14:** AUC (Mean ± Standard Deviation) Table of Segmentation Output on Test Set in the Target Domain (Domain Adaptation)

AUC (%)	$w_{0,1}=12.0$	$w_{0,1}=6.0$	$w_{0,1}=3.0$	$w_{0,1}=1.5$	$w_{0,1}=1.0$	$w_{0,1}=1.0$	$w_{0,1}=1.0$	$w_{0,1}=1.0$	$w_{0,1}=1.0$
	$w_{2}=1.0$	$w_{2}=1.0$	$w_{2}=1.0$	$w_{2}=1.0$	$w_{2}=1.0$	$w_{2}=1.5$	$w_{2}=3.0$	$w_{2}=6.0$	$w_{2}=12.0$
$T^{2}=0.40$	51.1±21.6	37.9±20.8	56.3±18.6	55.0±17.3	82.6±5.6	90.0±3.6	95.9±0.7	97.5±0.9	95.8±1.5
$T^{2}=0.20$	47.0±19.7	41.2±20.9	76.5±9.6	20.4±3.9	52.3±10.7	94.5±2.1	98.0±0.4	98.2±0.7	92.8±3.9
$T^{2}=0.15$	53.9±26.7	56.5±19.0	28.6±6.9	31.9±3.0	44.5±3.0	93.0±7.0	97.4±0.8	96.4±2.9	95.7±1.7
$T^{2}=0.10$	46.4±10.9	70.8±13.3	19.5±6.4	21.0±3.0	59.6±20.5	95.6±1.3	97.0±0.4	97.2±1.0	90.2±8.3
$T^{2}=0.05$	62.4±10.3	37.3±16.6	16.3±2.1	22.5±5.7	60.4±7.4	97.0±0.9	96.0±1.6	96.5±1.5	93.1±1.2
$T^{2}=0.01$	33.6±4.3	17.3±2.9	13.7±2.8	19.4±8.8	61.6±17.8	92.5±7.6	96.6±0.8	97.2±1.3	92.0±4.8
$T^{2}=0.00$	44.0±6.4	16.4±3.1	12.6±2.4	24.8±6.2	63.8±13.4	97.0±0.5	97.1±0.7	97.8±0.4	90.9±4.9

**TABLE 15:** F1-Score (Mean ± Standard Deviation) Table of Segmentation Output on Test Set in the Target Domain (Domain Adaptation)

F1-score (%)	$w_{0,1}=12.0$	$w_{0,1}=6.0$	$w_{0,1}=3.0$	$w_{0,1}=1.5$	$w_{0,1}=1.0$	$w_{0,1}=1.0$	$w_{0,1}=1.0$	$w_{0,1}=1.0$	$w_{0,1}=1.0$
	$w_{2}=1.0$	$w_{2}=1.0$	$w_{2}=1.0$	$w_{2}=1.0$	$w_{2}=1.0$	$w_{2}=1.5$	$w_{2}=3.0$	$w_{2}=6.0$	$w_{2}=12.0$
$T^{2}=0.40$	0.0±0.0	0.0±0.0	0.0±0.0	0.0±0.0	2.5±2.6	8.9±8.8	74.5±4.9	93.4±1.9	93.5±1.3
$T^{2}=0.20$	0.0±0.0	0.0±0.0	1.3±0.5	6.1±0.3	49.5±8.1	89.4±3.2	95.5±0.7	95.9±0.5	91.7±2.3
$T^{2}=0.15$	0.0±0.0	0.0±0.0	1.9±1.5	31.9±2.8	59.4±2.0	86.7±9.8	95.0±0.6	94.3±2.5	94.4±1.1
$T^{2}=0.10$	0.0±0.0	0.0±0.0	9.8±3.3	38.1±3.7	68.7±8.4	83.8±4.4	94.0±0.6	95.0±0.4	89.7±5.8
$T^{2}=0.05$	0.0±0.0	9.7±4.2	36.5±4.1	57.5±4.5	67.4±1.1	88.6±4.3	94.1±0.8	93.8±1.4	93.0±0.7
$T^{2}=0.01$	60.9±1.4	63.1±1.0	58.6±0.9	59.6±4.5	70.1±5.3	86.4±8.7	94.3±0.7	94.7±0.9	88.9±6.6
$T^{2}=0.00$	66.5±0.1	64.2±0.8	56.3±1.5	62.9±2.6	68.5±2.1	90.0±1.8	94.8±0.5	95.1±0.6	87.4±9.4

**TABLE 16:** Accuracy (Mean ± Standard Deviation) Table of Classification Output on Validation Set in the Target Domain (No Domain Adaptation)

Accuracy (%)	$w_{0,1}=12.0$	$w_{0,1}=6.0$	$w_{0,1}=3.0$	$w_{0,1}=1.5$	$w_{0,1}=1.0$	$w_{0,1}=1.0$	$w_{0,1}=1.0$	$w_{0,1}=1.0$	$w_{0,1}=1.0$
	$w_{2}=1.0$	$w_{2}=1.0$	$w_{2}=1.0$	$w_{2}=1.0$	$w_{2}=1.0$	$w_{2}=1.5$	$w_{2}=3.0$	$w_{2}=6.0$	$w_{2}=12.0$
$T^{2}=0.40$	50.0±0.0	50.0±0.0	50.0±0.0	50.2±0.4	50.0±0.0	52.7±2.2	74.8±11.8	75.6±3.3	64.4±3.3
$T^{2}=0.20$	50.0±0.0	50.0±0.0	50.2±0.4	56.0±8.5	44.6±8.2	53.1±3.2	84.5±4.0	78.1±3.9	60.7±0.9
$T^{2}=0.15$	50.0±0.0	50.0±0.0	50.9±0.9	38.3±8.4	38.7±2.8	65.0±9.4	86.1±5.0	74.9±4.9	60.7±3.6
$T^{2}=0.10$	50.0±0.0	50.0±0.0	58.3±2.8	36.7±5.2	45.4±4.0	63.7±6.3	87.4±5.2	74.9±5.0	58.8±2.5
$T^{2}=0.05$	50.0±0.0	57.7±3.2	37.0±8.2	31.3±4.0	40.1±2.2	67.2±2.5	84.5±3.9	73.9±5.0	62.0±3.7
$T^{2}=0.01$	53.0±5.1	56.6±7.3	33.1±2.7	30.5±5.7	38.4±1.7	69.1±3.0	82.7±6.8	73.7±5.0	59.8±5.2
$T^{2}=0.00$	48.0±13.0	55.9±6.0	30.8±4.0	30.6±4.0	41.9±3.8	60.9±7.6	84.2±3.2	76.0±2.8	62.2±3.4

**TABLE 17:** AUC (Mean ± Standard Deviation) Table of Classification Output on Validation Set in the Target Domain (No Domain Adaptation)

AUC (%)	$w_{0,1}=12.0$	$w_{0,1}=6.0$	$w_{0,1}=3.0$	$w_{0,1}=1.5$	$w_{0,1}=1.0$	$w_{0,1}=1.0$	$w_{0,1}=1.0$	$w_{0,1}=1.0$	$w_{0,1}=1.0$
	$w_{2}=1.0$	$w_{2}=1.0$	$w_{2}=1.0$	$w_{2}=1.0$	$w_{2}=1.0$	$w_{2}=1.5$	$w_{2}=3.0$	$w_{2}=6.0$	$w_{2}=12.0$
$T^{2}=0.40$	39.8±9.7	45.5±13.3	30.2±12.8	66.6±14.7	78.3±8.6	74.0±12.5	85.5±8.3	80.1±5.2	73.7±10.0
$T^{2}=0.20$	34.7±15.6	35.0±9.1	71.8±4.3	55.6±14.6	57.6±7.3	74.2±3.7	93.2±1.9	83.5±8.4	78.8±9.1
$T^{2}=0.15$	43.9±16.2	68.5±18.9	40.7±5.0	45.4±6.9	44.0±2.8	77.0±4.0	91.3±4.2	85.4±6.1	81.3±10.2
$T^{2}=0.10$	16.8±9.6	58.5±21.0	62.9±6.3	36.8±4.8	52.6±6.2	74.7±1.9	90.9±4.4	82.5±10.7	77.8±12.2
$T^{2}=0.05$	56.3±6.7	74.1±1.2	34.2±7.8	30.4±4.2	46.8±5.1	75.9±4.4	90.2±4.2	87.2±2.2	83.4±9.6
$T^{2}=0.01$	48.9±11.1	50.5±13.0	28.5±5.2	32.0±4.0	43.5±7.0	74.3±4.6	90.6±4.9	86.6±2.7	83.9±3.3
$T^{2}=0.00$	38.2±13.6	51.5±9.5	27.3±4.8	32.4±6.4	48.4±2.7	72.0±2.8	90.9±4.3	85.6±7.9	80.2±5.5

**TABLE 18:** F1-Score (Mean ± Standard Deviation) Table of Classification Output on Validation Set in the Target Domain (No Domain Adaptation)

F1-score (%)	$w_{0,1}=12.0$	$w_{0,1}=6.0$	$w_{0,1}=3.0$	$w_{0,1}=1.5$	$w_{0,1}=1.0$	$w_{0,1}=1.0$	$w_{0,1}=1.0$	$w_{0,1}=1.0$	$w_{0,1}=1.0$
	$w_{2}=1.0$	$w_{2}=1.0$	$w_{2}=1.0$	$w_{2}=1.0$	$w_{2}=1.0$	$w_{2}=1.5$	$w_{2}=3.0$	$w_{2}=6.0$	$w_{2}=12.0$
$T^{2}=0.40$	0.0±0.0	0.0±0.0	0.0±0.0	1.0±1.7	0.0±0.0	9.9±7.6	75.5±9.8	69.4±5.3	44.3±7.8
$T^{2}=0.20$	0.0±0.0	0.0±0.0	1.0±1.7	44.6±14.4	56.1±4.7	64.5±2.5	84.2±4.2	72.1±6.4	35.4±2.4
$T^{2}=0.15$	0.0±0.0	0.0±0.0	3.5±3.5	48.7±5.9	53.0±3.7	69.2±4.8	85.8±5.1	66.4±9.4	34.5±10.1
$T^{2}=0.10$	0.0±0.0	0.0±0.0	35.7±9.8	50.2±4.5	55.8±1.5	67.4±2.7	86.1±6.6	67.3±8.1	29.6±7.4
$T^{2}=0.05$	0.0±0.0	27.3±9.6	41.4±8.8	42.8±4.1	52.3±2.5	68.9±3.5	83.1±4.5	64.0±10.0	38.2±9.5
$T^{2}=0.01$	25.4±17.8	40.2±2.8	35.4±5.7	40.7±4.5	48.6±2.4	68.5±2.8	82.0±6.4	64.2±10.1	31.6±13.9
$T^{2}=0.00$	24.1±15.4	42.3±7.8	31.1±6.2	40.3±4.1	51.6±3.6	64.3±3.7	82.7±4.3	68.4±4.9	38.8±8.7

**TABLE 19:** Accuracy (Mean ± Standard Deviation) Table of Segmentation Output on Validation Set in the Target Domain (No Domain Adaptation)

Accuracy (%)	$w_{0,1}=12.0$	$w_{0,1}=6.0$	$w_{0,1}=3.0$	$w_{0,1}=1.5$	$w_{0,1}=1.0$	$w_{0,1}=1.0$	$w_{0,1}=1.0$	$w_{0,1}=1.0$	$w_{0,1}=1.0$
	$w_{2}=1.0$	$w_{2}=1.0$	$w_{2}=1.0$	$w_{2}=1.0$	$w_{2}=1.0$	$w_{2}=1.5$	$w_{2}=3.0$	$w_{2}=6.0$	$w_{2}=12.0$
$T^{2}=0.40$	50.0±0.0	50.0±0.0	50.0±0.0	50.0±0.0	50.0±0.0	51.8±2.5	59.1±10.7	51.9±6.0	53.1±8.8
$T^{2}=0.20$	50.0±0.0	50.0±0.0	50.8±1.3	53.7±3.2	42.3±9.1	42.2±3.5	61.3±9.5	53.9±4.4	58.6±10.6
$T^{2}=0.15$	50.0±0.0	50.0±0.0	50.0±0.0	31.0±4.3	33.2±2.7	46.0±1.4	55.6±4.8	58.6±6.1	65.2±7.6
$T^{2}=0.10$	50.0±0.0	50.0±0.0	54.0±2.6	29.0±5.3	38.3±3.9	46.4±0.9	53.9±4.6	54.4±5.3	62.7±10.8
$T^{2}=0.05$	50.0±0.0	53.8±1.8	26.9±5.5	33.3±2.0	41.8±3.0	46.1±1.5	57.7±3.0	61.0±4.0	67.0±9.8
$T^{2}=0.01$	47.4±1.7	40.9±4.2	31.5±4.1	34.9±3.7	40.9±2.4	46.6±0.4	55.9±2.8	53.6±4.4	62.0±10.8
$T^{2}=0.00$	49.8±0.3	39.0±4.7	31.6±2.7	34.3±3.1	40.6±3.4	46.4±1.6	54.7±5.4	57.2±6.4	57.3±10.3

**TABLE 20:** AUC (Mean ± Standard Deviation) Table of Segmentation Output on Validation Set in the Target Domain (No Domain Adaptation)

AUC (%)	$w_{0,1}=12.0$	$w_{0,1}=6.0$	$w_{0,1}=3.0$	$w_{0,1}=1.5$	$w_{0,1}=1.0$	$w_{0,1}=1.0$	$w_{0,1}=1.0$	$w_{0,1}=1.0$	$w_{0,1}=1.0$
	$w_{2}=1.0$	$w_{2}=1.0$	$w_{2}=1.0$	$w_{2}=1.0$	$w_{2}=1.0$	$w_{2}=1.5$	$w_{2}=3.0$	$w_{2}=6.0$	$w_{2}=12.0$
$T^{2}=0.40$	39.7±7.4	37.3±9.5	34.4±5.8	60.1±24.4	60.6±24.4	69.2±10.0	65.9±8.0	68.2±7.2	57.2±9.0
$T^{2}=0.20$	40.3±10.3	42.8±13.4	68.1±21.5	48.0±4.5	39.1±10.6	43.0±5.8	86.5±7.4	77.5±7.7	64.5±11.0
$T^{2}=0.15$	46.9±10.0	57.9±20.1	61.5±12.8	25.6±4.8	28.6±5.3	52.1±10.5	85.0±7.5	78.0±8.2	70.8±9.8
$T^{2}=0.10$	33.7±8.1	52.4±25.7	48.5±7.8	26.9±10.7	34.9±5.6	56.4±5.5	84.7±6.8	75.8±10.5	68.8±13.2
$T^{2}=0.05$	62.7±15.0	60.8±2.6	21.6±6.1	23.8±1.8	36.4±2.0	58.9±2.2	85.7±6.5	81.7±3.8	73.2±9.6
$T^{2}=0.01$	44.3±5.4	40.0±10.4	20.8±4.5	21.9±5.0	32.0±3.7	64.3±4.8	86.8±5.7	76.0±8.5	68.2±8.7
$T^{2}=0.00$	55.2±8.7	38.6±8.5	18.9±2.5	22.2±5.1	35.9±4.0	60.0±2.0	85.2±6.3	77.7±8.7	65.2±9.6

**TABLE 21:** F1-Score (Mean ± Standard Deviation) Table of Segmentation Output on Validation Set in the Target Domain (No Domain Adaptation)

F1-score (%)	$w_{0,1}=12.0$	$w_{0,1}=6.0$	$w_{0,1}=3.0$	$w_{0,1}=1.5$	$w_{0,1}=1.0$	$w_{0,1}=1.0$	$w_{0,1}=1.0$	$w_{0,1}=1.0$	$w_{0,1}=1.0$
	$w_{2}=1.0$	$w_{2}=1.0$	$w_{2}=1.0$	$w_{2}=1.0$	$w_{2}=1.0$	$w_{2}=1.5$	$w_{2}=3.0$	$w_{2}=6.0$	$w_{2}=12.0$
$T^{2}=0.40$	0.0±0.0	0.0±0.0	0.0±0.0	0.0±0.0	0.0±0.0	7.2±8.6	61.7±7.9	62.9±3.9	58.1±6.4
$T^{2}=0.20$	0.0±0.0	0.0±0.0	2.9±5.0	18.1±7.5	46.9±6.0	57.0±1.1	71.7±5.4	65.8±3.3	62.6±8.7
$T^{2}=0.15$	0.0±0.0	0.0±0.0	0.0±0.0	34.7±6.2	48.5±3.9	62.2±1.5	68.3±2.5	69.3±3.9	65.0±9.1
$T^{2}=0.10$	0.0±0.0	0.0±0.0	21.5±8.8	41.6±3.4	54.2±5.0	62.8±0.6	67.3±2.9	66.0±5.0	63.4±10.3
$T^{2}=0.05$	0.0±0.0	18.7±3.6	35.1±7.9	49.3±2.8	58.4±3.5	63.0±1.5	69.3±1.1	69.6±1.6	67.0±9.2
$T^{2}=0.01$	64.3±1.6	52.0±4.1	44.9±6.1	50.9±4.5	57.6±2.9	62.9±0.6	68.8±1.4	65.1±5.0	62.6±8.9
$T^{2}=0.00$	66.5±0.3	53.3±5.3	45.9±4.1	50.2±4.0	57.2±4.0	63.2±1.7	67.9±2.9	68.1±3.9	61.6±9.6

**TABLE 22:** Accuracy (Mean ± Standard Deviation) Table of Classification Output on Test Set in the Target Domain (No Domain Adaptation)

Accuracy (%)	$w_{0,1}=12.0$	$w_{0,1}=6.0$	$w_{0,1}=3.0$	$w_{0,1}=1.5$	$w_{0,1}=1.0$	$w_{0,1}=1.0$	$w_{0,1}=1.0$	$w_{0,1}=1.0$	$w_{0,1}=1.0$
	$w_{2}=1.0$	$w_{2}=1.0$	$w_{2}=1.0$	$w_{2}=1.0$	$w_{2}=1.0$	$w_{2}=1.5$	$w_{2}=3.0$	$w_{2}=6.0$	$w_{2}=12.0$
$T^{2}=0.40$	50.0±0.0	50.0±0.0	50.0±0.0	50.0±0.0	50.0±0.0	58.1±5.8	79.0±9.4	81.5±2.3	60.1±1.6
$T^{2}=0.20$	50.0±0.0	50.0±0.0	50.0±0.1	59.5±6.2	53.5±7.6	57.7±2.1	87.6±2.8	85.0±2.8	57.2±1.4
$T^{2}=0.15$	50.0±0.0	50.0±0.1	50.4±0.5	46.8±6.0	46.3±2.2	71.5±8.3	88.7±2.6	80.4±3.7	57.4±2.5
$T^{2}=0.10$	50.0±0.0	50.0±0.0	56.8±2.7	41.1±1.9	52.9±3.7	70.3±5.5	89.4±2.7	81.2±4.6	56.6±1.1
$T^{2}=0.05$	50.0±0.0	55.9±1.0	36.9±4.7	36.7±2.0	50.6±2.6	75.9±2.3	89.6±1.3	77.3±5.4	59.1±2.2
$T^{2}=0.01$	51.8±4.0	53.4±6.3	32.4±2.7	36.0±3.2	48.5±2.7	79.2±2.9	86.4±7.4	77.5±3.9	57.7±5.1
$T^{2}=0.00$	49.2±9.1	53.3±4.5	31.7±3.3	36.6±2.0	49.9±3.8	72.2±7.7	87.7±4.4	79.5±1.6	60.2±3.8

**TABLE 23:** AUC (Mean ± Standard Deviation) Table of Classification Output on Test Set in the Target Domain (No Domain Adaptation)

AUC (%)	$w_{0,1}=12.0$	$w_{0,1}=6.0$	$w_{0,1}=3.0$	$w_{0,1}=1.5$	$w_{0,1}=1.0$	$w_{0,1}=1.0$	$w_{0,1}=1.0$	$w_{0,1}=1.0$	$w_{0,1}=1.0$
	$w_{2}=1.0$	$w_{2}=1.0$	$w_{2}=1.0$	$w_{2}=1.0$	$w_{2}=1.0$	$w_{2}=1.5$	$w_{2}=3.0$	$w_{2}=6.0$	$w_{2}=12.0$
$T^{2}=0.40$	41.2±15.5	48.2±20.2	34.4±18.7	62.7±17.4	72.7±6.9	84.0±9.1	89.3±3.5	86.3±4.5	81.9±3.0
$T^{2}=0.20$	37.3±24.5	35.4±18.6	66.1±7.7	60.9±10.0	72.8±4.5	85.7±3.0	94.3±0.6	91.5±2.6	84.8±6.4
$T^{2}=0.15$	48.4±17.7	74.0±11.6	39.9±8.7	54.5±8.1	55.8±6.3	88.1±1.4	94.2±1.0	90.5±3.6	87.8±4.8
$T^{2}=0.10$	22.7±13.3	54.6±18.1	55.3±8.2	40.8±3.5	64.5±5.3	86.4±2.5	94.0±1.2	90.1±2.9	83.5±7.6
$T^{2}=0.05$	61.7±12.8	69.0±3.3	35.1±4.6	32.7±4.4	57.8±6.6	89.6±0.7	94.2±1.0	91.7±1.8	87.3±5.6
$T^{2}=0.01$	41.2±12.0	46.0±7.5	28.6±3.9	34.4±4.5	54.6±2.8	88.5±2.4	93.7±2.6	90.7±1.8	87.1±1.2
$T^{2}=0.00$	38.6±11.5	47.9±4.4	28.4±4.6	34.8±3.1	58.9±7.5	86.6±2.4	94.3±1.3	90.8±2.6	85.0±3.6

**TABLE 24:** F1-Score (Mean ± Standard Deviation) Table of Classification Output on Test Set in the Target Domain (No Domain Adaptation)

F1-score (%)	$w_{0,1}=12.0$	$w_{0,1}=6.0$	$w_{0,1}=3.0$	$w_{0,1}=1.5$	$w_{0,1}=1.0$	$w_{0,1}=1.0$	$w_{0,1}=1.0$	$w_{0,1}=1.0$	$w_{0,1}=1.0$
	$w_{2}=1.0$	$w_{2}=1.0$	$w_{2}=1.0$	$w_{2}=1.0$	$w_{2}=1.0$	$w_{2}=1.5$	$w_{2}=3.0$	$w_{2}=6.0$	$w_{2}=12.0$
$T^{2}=0.40$	0.0±0.0	0.0±0.0	0.0±0.0	0.1±0.2	0.0±0.0	26.4±16.5	80.9±6.4	79.3±1.9	34.4±4.5
$T^{2}=0.20$	0.0±0.0	0.0±0.0	0.2±0.3	49.7±9.0	64.7±3.7	68.7±1.0	87.9±2.1	83.0±3.6	25.8±4.7
$T^{2}=0.15$	0.0±0.0	0.2±0.3	1.7±2.0	57.1±3.4	60.5±2.4	76.0±4.9	88.9±2.2	76.0±5.6	25.7±7.5
$T^{2}=0.10$	0.0±0.0	0.0±0.0	32.9±2.6	53.9±1.6	63.0±1.0	74.8±3.3	89.4±2.4	77.3±6.9	24.1±3.3
$T^{2}=0.05$	0.0±0.0	22.2±4.0	39.8±2.4	47.9±1.9	61.7±1.6	78.8±1.2	89.5±1.2	70.4±8.9	30.9±5.9
$T^{2}=0.01$	23.5±12.3	35.9±3.8	33.0±4.7	45.7±2.6	59.0±1.3	80.4±2.4	87.2±5.8	71.4±6.7	26.1±14.3
$T^{2}=0.00$	22.0±18.6	39.8±4.0	33.1±2.9	46.4±2.0	60.4±3.4	76.1±4.7	88.0±3.5	74.7±2.4	34.0±10.2

**TABLE 25:** Accuracy (Mean ± Standard Deviation) Table of Segmentation Output on Test Set in the Target Domain (No Domain Adaptation)

Accuracy (%)	$w_{0,1}=12.0$	$w_{0,1}=6.0$	$w_{0,1}=3.0$	$w_{0,1}=1.5$	$w_{0,1}=1.0$	$w_{0,1}=1.0$	$w_{0,1}=1.0$	$w_{0,1}=1.0$	$w_{0,1}=1.0$
	$w_{2}=1.0$	$w_{2}=1.0$	$w_{2}=1.0$	$w_{2}=1.0$	$w_{2}=1.0$	$w_{2}=1.5$	$w_{2}=3.0$	$w_{2}=6.0$	$w_{2}=12.0$
$T^{2}=0.40$	50.0±0.0	50.0±0.0	50.0±0.0	50.0±0.0	50.1±0.2	51.6±1.5	65.9±8.4	56.6±6.0	59.5±4.9
$T^{2}=0.20$	50.0±0.0	50.0±0.0	51.0±1.7	55.9±4.1	47.4±7.2	50.3±2.9	60.7±7.8	58.7±3.4	63.4±7.0
$T^{2}=0.15$	50.0±0.0	50.0±0.0	50.0±0.1	36.8±1.9	43.6±1.7	52.0±2.2	56.0±2.4	59.0±5.5	72.8±3.6
$T^{2}=0.10$	50.0±0.0	50.0±0.1	51.3±4.0	38.8±3.0	49.2±2.1	50.7±1.0	54.9±1.6	58.0±1.9	69.7±7.6
$T^{2}=0.05$	50.1±0.1	53.5±1.3	29.5±1.1	41.4±1.1	48.9±0.4	50.1±0.4	57.2±3.7	62.8±4.3	73.4±6.6
$T^{2}=0.01$	50.0±0.0	40.8±1.9	33.3±2.6	42.0±1.7	48.3±0.9	51.3±1.1	56.9±3.9	57.1±3.2	70.2±5.2
$T^{2}=0.00$	50.0±0.0	41.1±1.0	34.3±1.0	42.6±1.5	48.7±0.7	50.2±0.7	55.3±4.0	60.0±5.1	62.7±7.7

**TABLE 26:** AUC (Mean ± Standard Deviation) Table of Segmentation Output on Test Set in the Target Domain (No Domain Adaptation)

AUC (%)	$w_{0,1}=12.0$	$w_{0,1}=6.0$	$w_{0,1}=3.0$	$w_{0,1}=1.5$	$w_{0,1}=1.0$	$w_{0,1}=1.0$	$w_{0,1}=1.0$	$w_{0,1}=1.0$	$w_{0,1}=1.0$
	$w_{2}=1.0$	$w_{2}=1.0$	$w_{2}=1.0$	$w_{2}=1.0$	$w_{2}=1.0$	$w_{2}=1.5$	$w_{2}=3.0$	$w_{2}=6.0$	$w_{2}=12.0$
$T^{2}=0.40$	32.2±5.8	34.3±9.3	29.5±4.6	52.1±21.2	57.5±20.4	77.5±8.5	76.0±3.9	78.5±3.7	67.1±5.4
$T^{2}=0.20$	31.3±5.8	34.6±11.1	66.8±18.3	54.3±7.5	49.8±8.1	59.0±8.4	91.4±2.2	84.5±3.0	74.5±8.0
$T^{2}=0.15$	40.3±7.8	49.9±26.5	55.6±7.8	32.9±2.8	39.5±5.4	69.8±11.5	91.0±1.9	83.3±5.7	80.7±4.0
$T^{2}=0.10$	32.5±7.4	54.6±23.2	47.0±9.8	33.2±9.4	47.8±8.3	72.2±7.7	91.0±1.3	82.5±3.7	77.6±7.9
$T^{2}=0.05$	76.5±12.1	61.4±5.5	23.3±1.5	24.6±3.5	45.6±3.5	77.8±4.0	91.0±1.4	86.4±1.3	80.8±4.7
$T^{2}=0.01$	45.3±11.8	39.1±7.6	20.6±1.8	23.4±2.2	42.5±4.8	81.4±3.8	90.8±3.2	82.5±2.0	77.5±3.8
$T^{2}=0.00$	53.9±8.7	38.6±4.5	20.8±2.2	22.8±2.3	45.7±6.7	75.4±5.6	91.3±2.1	84.4±3.7	74.2±4.2

**TABLE 27:** F1-Score (Mean ± Standard Deviation) Table of Segmentation Output on Test Set in the Target Domain (No Domain Adaptation)

F1-score (%)	$w_{0,1}=12.0$	$w_{0,1}=6.0$	$w_{0,1}=3.0$	$w_{0,1}=1.5$	$w_{0,1}=1.0$	$w_{0,1}=1.0$	$w_{0,1}=1.0$	$w_{0,1}=1.0$	$w_{0,1}=1.0$
	$w_{2}=1.0$	$w_{2}=1.0$	$w_{2}=1.0$	$w_{2}=1.0$	$w_{2}=1.0$	$w_{2}=1.5$	$w_{2}=3.0$	$w_{2}=6.0$	$w_{2}=12.0$
$T^{2}=0.40$	0.0±0.0	0.0±0.0	0.0±0.0	0.0±0.0	0.5±0.6	6.2±5.4	69.3±4.6	67.3±3.2	64.5±3.3
$T^{2}=0.20$	0.0±0.0	0.0±0.0	3.8±6.3	22.5±12.7	53.0±2.3	65.2±1.7	71.5±4.0	69.8±1.9	68.5±5.3
$T^{2}=0.15$	0.0±0.0	0.0±0.0	0.2±0.3	39.1±6.5	59.1±1.8	67.0±1.1	68.9±1.1	69.8±3.0	74.6±2.9
$T^{2}=0.10$	0.0±0.0	0.2±0.3	17.0±8.2	51.7±2.5	64.4±1.0	66.6±0.4	68.3±0.8	69.2±0.9	72.4±5.4
$T^{2}=0.05$	0.4±0.5	19.2±6.0	35.5±3.5	57.1±1.4	65.1±0.5	66.3±0.1	69.5±1.8	71.5±2.2	75.3±4.9
$T^{2}=0.01$	66.7±0.0	50.7±2.5	46.0±4.2	57.5±1.9	64.4±0.6	66.9±0.6	69.3±2.1	68.7±1.4	72.4±3.2
$T^{2}=0.00$	66.7±0.0	53.7±2.6	47.6±1.0	58.3±1.5	64.8±0.8	66.4±0.4	68.6±1.8	70.4±2.5	68.8±4.2

#### 1) Standard DRRs Versus Infection-Aware DRRs

**FIGURE 7.**
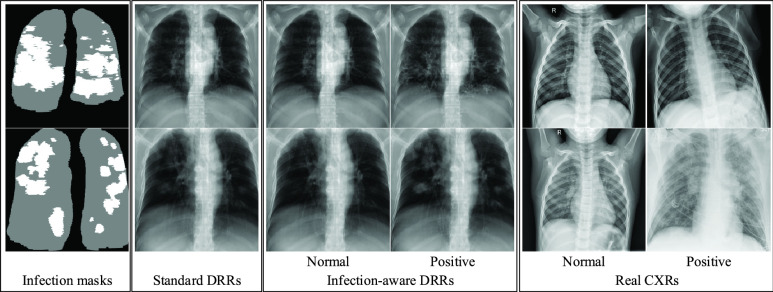
Comparison of standard DRRs, infection-aware DRRs, and real CXRs. The first column represents the infection masks that are generated with the contribution threshold $T^{1}=T^{2}=0.00$. We use such infection masks to indicate the infected pixels of DRRs whose corresponding X-rays pass through the infected voxels of CT volume.

Firstly, we do qualitative comparison in Fig. 7. As can be seen, many infected pixels in standard DRRs indicated by infection masks present no-findings due to the low contribution rate of infected voxels in the X-ray casting. This observation is consistent with the heterogeneous nature of X-ray imaging, and implicates that X-ray imaging has a lower sensitivity than CT imaging. We notice that the radiological signs of COVID-19 infection in standard DRRs are indistinctive. The strength of radiological signs of COVID-19 infection in standard DRRs depends on the severity of COVID-19 infection. Such property makes it hard to leverage the publicly available CT volumes, because there is no guarantee that a positive CT scan will yield positive DRRs. In contrast, our infection-aware DRR generator is able to produce DRRs with different strengths of radiological signs of COVID-19 infection simply by adjusting the weight of infected voxels $w_{2}$. For instance, a CT case with mild COVID-19 infection can yield DRRs with significant radiological signs of COVID-19 infection; whereas a CT case with severe COVID-19 infection can yield normal DRRs. Seen from the last column in Fig. 6, the radiological signs of COVID-19 infection become more significant gradually as the weight of infected voxels $w_{2}$ increases. Such ability of our infection-aware DRR generator promotes to take full advantages of the publicly available CT volumes and determine the precise infection annotation masks for training infection segmentation models. Note that visual findings of COVID-19 infection in DRRs will be unrealistic when the value of $w_{2}$ is too large, e.g., $w_{2}=12.0$, which may break the correlation between real CXRs and synthetic DRRs.

**FIGURE 8.**
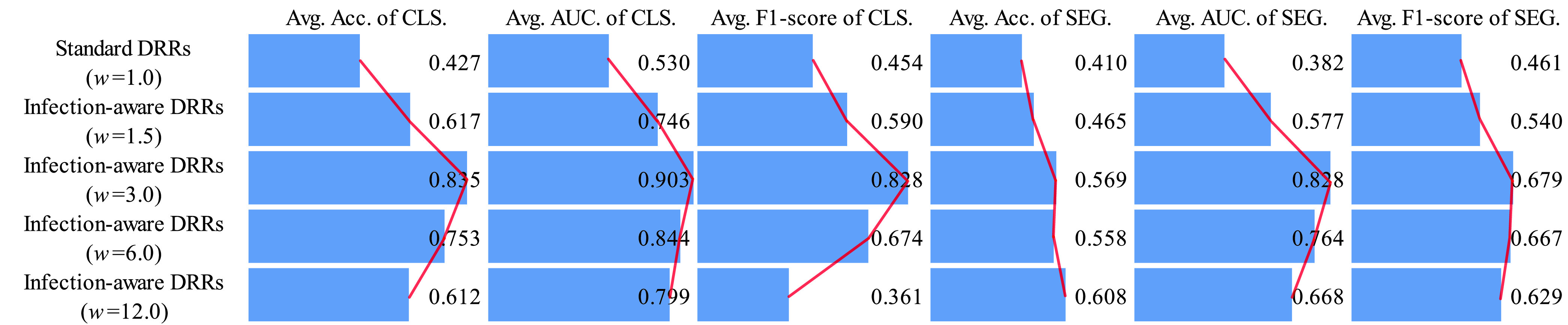
Comparison of average scores on the validation set in the target domain (no domain adaptation). CLS denotes classification results, SEG denotes segmentation results, and $w$ represents the weight of infected voxels $w_{2}$. The scores are averaged on CTIV ($T^{2}=0.40, 0.20, 0.15, 0.10, 0.05, 0.01, 0.00$).

**FIGURE 9.**
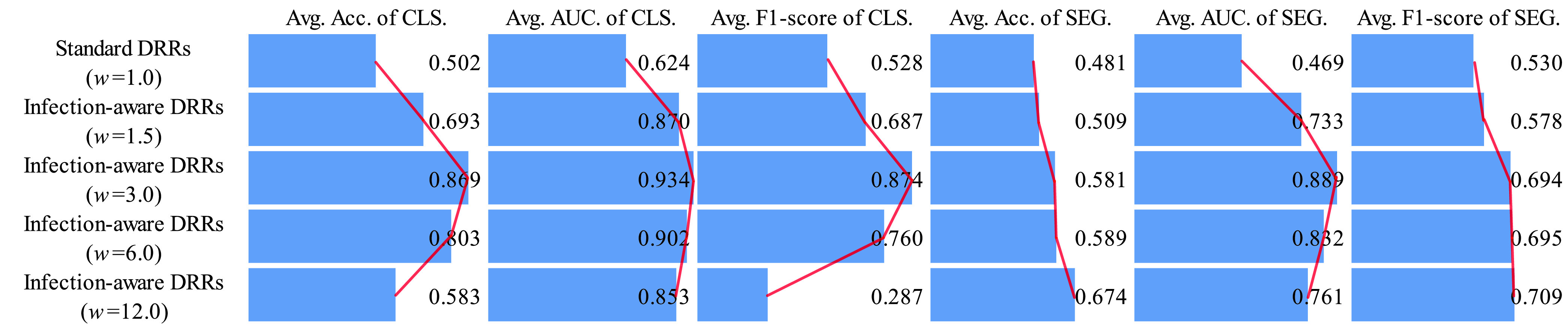
Comparison of average scores on the test set in the target domain (no domain adaptation). CLS denotes classification results, SEG denotes segmentation results, and $w$ represents the weight of infected voxels $w_{2}$. The scores are averaged on CTIV ($T^{2}=0.40,0.20,0.15,0.10,0.05,0.01,0.00$).

Secondly, we analyze the classification and segmentation results on the validation and test sets without using domain adaptation in Table 16–27 of the appendix. To avoid the influence of the subjective choice of contribution threshold, we average the performance scores on CTIV, and compare the average scores of standard DRRs and infection-aware DRRs visually in Fig. 8 and Fig. 9. Our infection-aware DRRs achieve significantly higher average scores on both validation and test sets in the target domain than the standard DRRs. Such results indicate that the gap between infection-aware DRRs (e.g., $w_{2}=3.0$) and real CXRs is smaller than the gap between standard DRRs ($w_{2}=1.0$) and real CXRs, and thus verify the efficacy of our infection-aware DRR generator without using domain adaptation.

**FIGURE 10.**
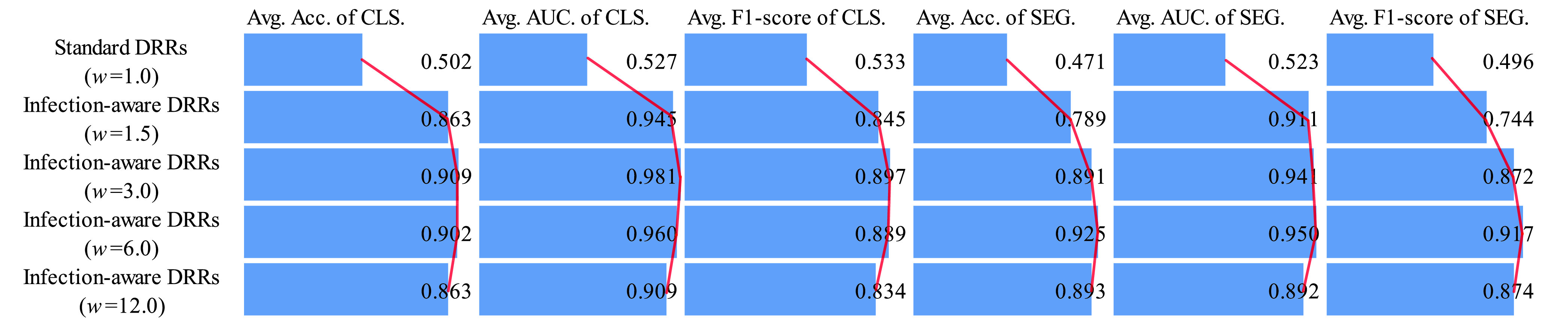
Comparison of average scores on the validation set in the target domain (domain adaptation). CLS denotes classification results, SEG denotes segmentation results, and $w$ represents the weight of infected voxels $w_{2}$. The scores are averaged on CTIV ($T^{2}=0.40,0.20,0.15,0.10,0.05,0.01,0.00$).

**FIGURE 11.**
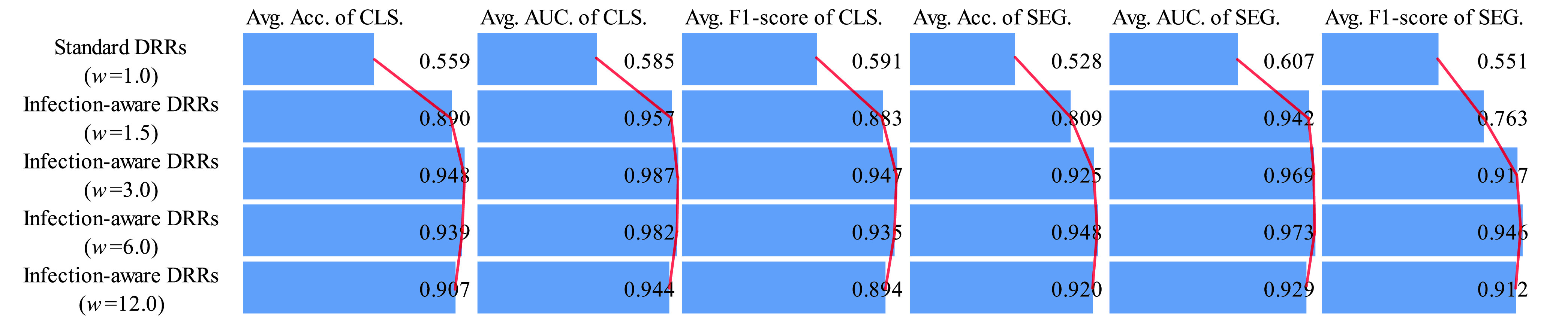
Comparison of average scores on the test set in the target domain (domain adaptation). CLS denotes classification results, SEG denotes segmentation results, and $w$ represents the weight of infected voxels $w_{2}$. The scores are averaged on CTIV ($T^{2}=0.40,0.20,0.15,0.10,0.05,0.01,0.00$).

Next, we analyze the classification and segmentation results on the validation and test sets with using domain adaptation in Table 4–15 of the appendix. We compare the average results of standard DRRs and infection-aware DRRs visually in Fig. 10 and Fig. 11. Similarly, the infection-aware DRRs surpass the standard DRRs by a large margin on both validation and test sets in the target domain. Such results strongly demonstrate the effectiveness of our infection-aware DRR generator with using the domain adaptation module.

Finally, we perform statistical tests to compare the means of scores in Fig. 8, Fig. 9, Fig. 10 and Fig. 11 between standard DRRs and infection-aware DRRs. We observe statistical significant difference (p ¡ 0.05) in all of these 96 comparison items, which indicates our infection-aware DRRs are significantly better than the standard DRRs in learning automated COVID-19 infection segmentation on CXRs from DRRs.

#### 2) Domain Adaptation Versus No Domain Adaptation

**FIGURE 12.**
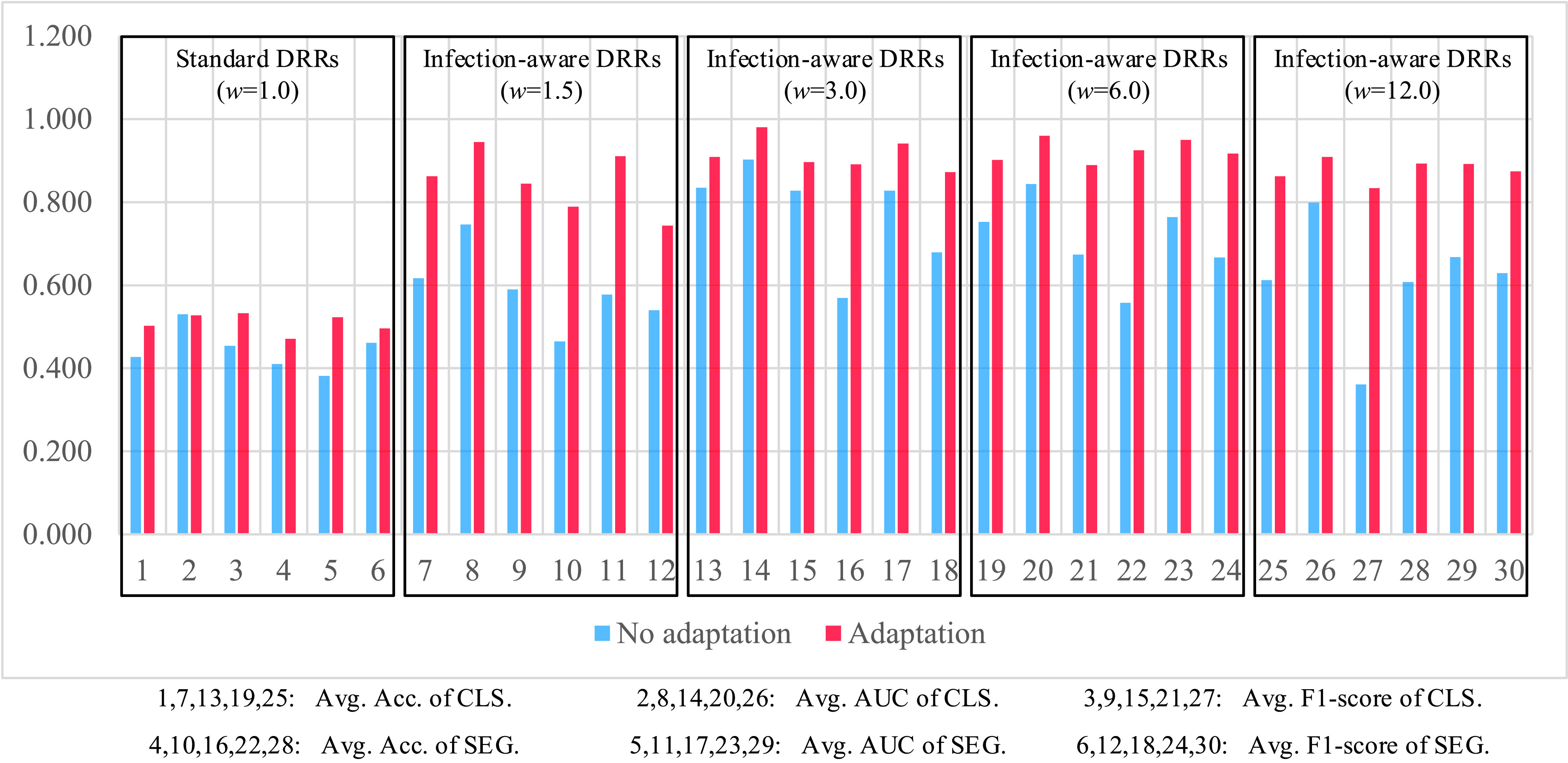
Comparison of average scores on the validation set with domain adaptation and without domain adaptation. The scores are averaged on CTIV ($T^{2}=0.40,0.20,0.15,0.10,0.05,0.01,0.00$).

**FIGURE 13.**
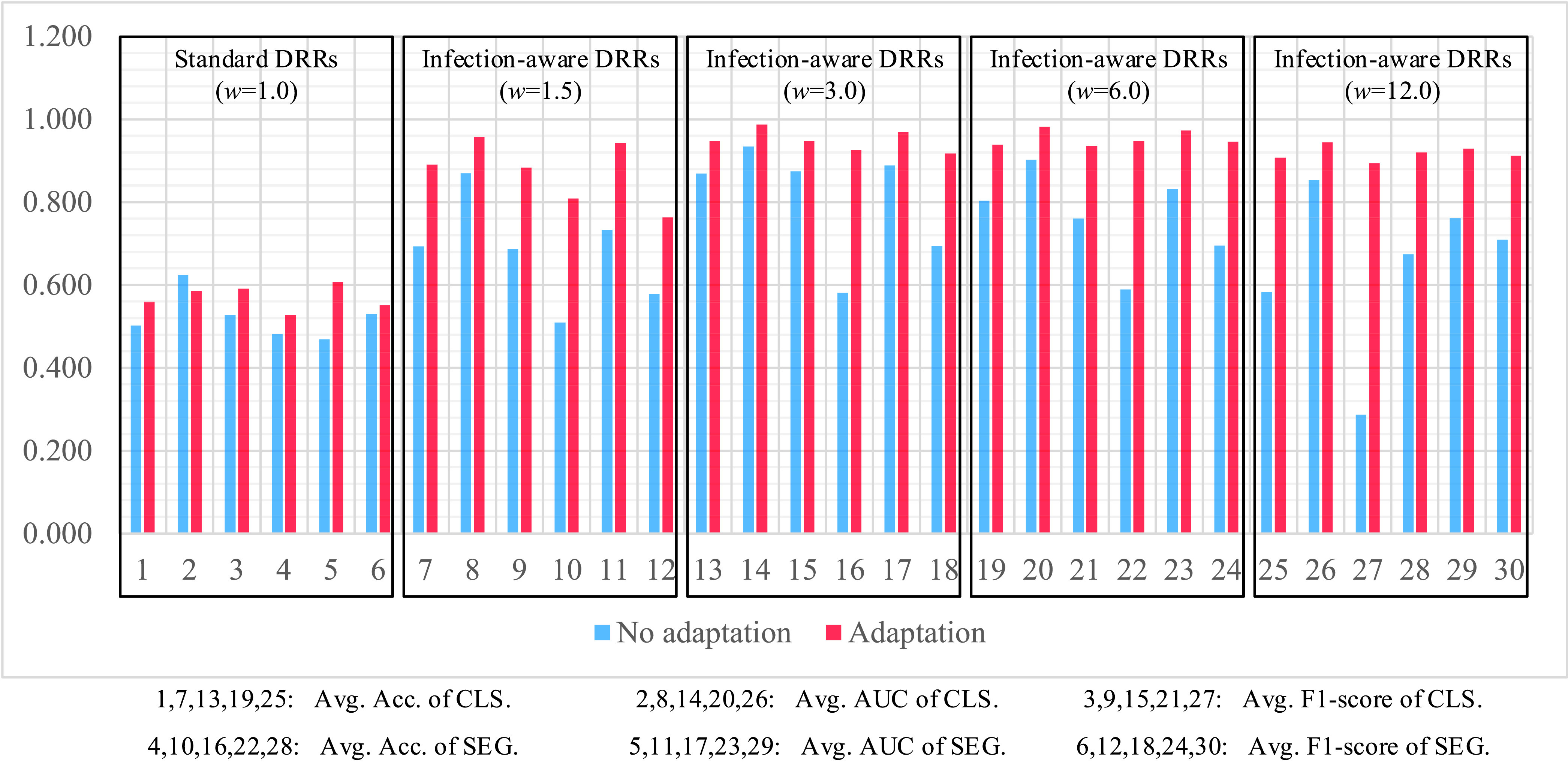
Comparison of average scores on the test set with domain adaptation and without domain adaptation. The scores are averaged on CTIV ($T^{2}=0.40,0.20,0.15,0.10,0.05,0.01,0.00$).

In order to highlight the efficacy of our domain adaptation module, we compare the average scores of domain adaptation and no domain adaptation on the validation and test sets in Fig. 12 and Fig. 13. This intuitive comparison shows that the using of our domain adaptation module can improve the classification and segmentation scores of infection-aware DRRs significantly and consistently, which verifies the efficacy of our domain adaptation module. Besides, seen from Fig. 8 and Fig. 9, we notice that the average scores of infection-aware DRRs increase first and then decrease as the weight of infected voxels $w_{2}$ increases from 1.0 to 3.0 and then to 12.0. The peak of average scores of infection-aware DRRs appears at $w_{2}=3.0$. It suggests that an excessively large weight of infected voxels may make the infected regions in DRRs unrealistic, thus leading to a decrease in performance scores without using domain adaptation module. In contrast, there is no significant decrease in the average scores of infection-aware DRRs with using our domain adaptation module as shown in Fig. 10 and Fig. 11 when the weight of infected voxels $w_{2}$ increases from 3.0 to 6.0 and then to 12.0. It implies that the domain adaptation module still works well even when infected regions in DRRs become slightly unrealistic. On the other hand, we observe that the segmentation scores are relatively lower than the classification scores when the domain adaptation module is not applied. For instance, in the case of infection-aware DRRs with $w_{2}=3.0$, the average segmentation scores on the test set in the target domain, including the accuracy, AUC and F1-score, are 0.581, 0.889, and 0.694 respectively, whereas the corresponding classification scores are 0.869, 0.934, and 0.874. Such results implicate that the segmentation header is much more sensitive to the domain discrepancy between DRRs and real CXRs than the classification header. By using the domain adaptation module, both the segmentation scores and classification scores are greatly improved; specifically, the improvement in segmentation scores is much more significant than the improvement in classification scores. For instance, in the case of infection-aware DRRs with $w_{2}=3.0$, the average segmentation scores on the test set are 0.925 ($+ 0.344\uparrow $), 0.969 ($+ 0.080\uparrow $), and 0.917 ($+ 0.223\uparrow $) respectively, whereas the corresponding classification scores are 0.948 ($+ 0.079\uparrow $), 0.987 ($+ 0.053\uparrow $), and 0.947 ($+ 0.073\uparrow $). Such results indicate that our domain adaptation module works well not only for classification task but also for segmentation task, thus confirming our claim that the effect of feature alignment applied in the classification branch can be propagated to the segmentation branch implicitly. Finally, we perform statistical tests to compare the means of scores in Fig. 12 and Fig. 13 between using our domain adaptation module and not using domain adaptation. We observe statistical significant difference (p ¡ 0.05) in all of these 60 comparison items except for 4 items, i.e., standard DRR’s classification AUC on the validation set and standard DRRs’ classification AUC, segmentation accuracy and segmentation F1-score on the test set. It indicates our domain adaptation module is able to improve the performance of learning infection segmentation on CXRs from DRRs significantly and thus confirms the efficacy of our domain adaptation module.

#### 3) Visualizing the COVID-19 Infection Segmentation Results

**FIGURE 14.**
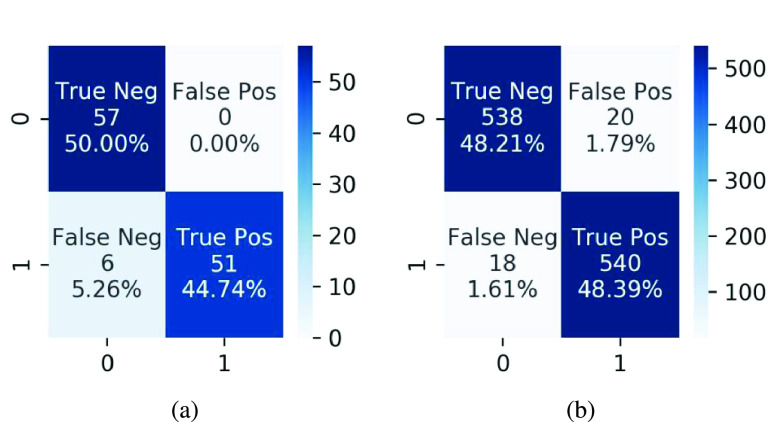
Confusion matrices of segmentation results on the validation and test sets in the case of infection-aware DRRs with $w_{2}=3.0$ and $T^{2}=0.20$.

**FIGURE 15.**
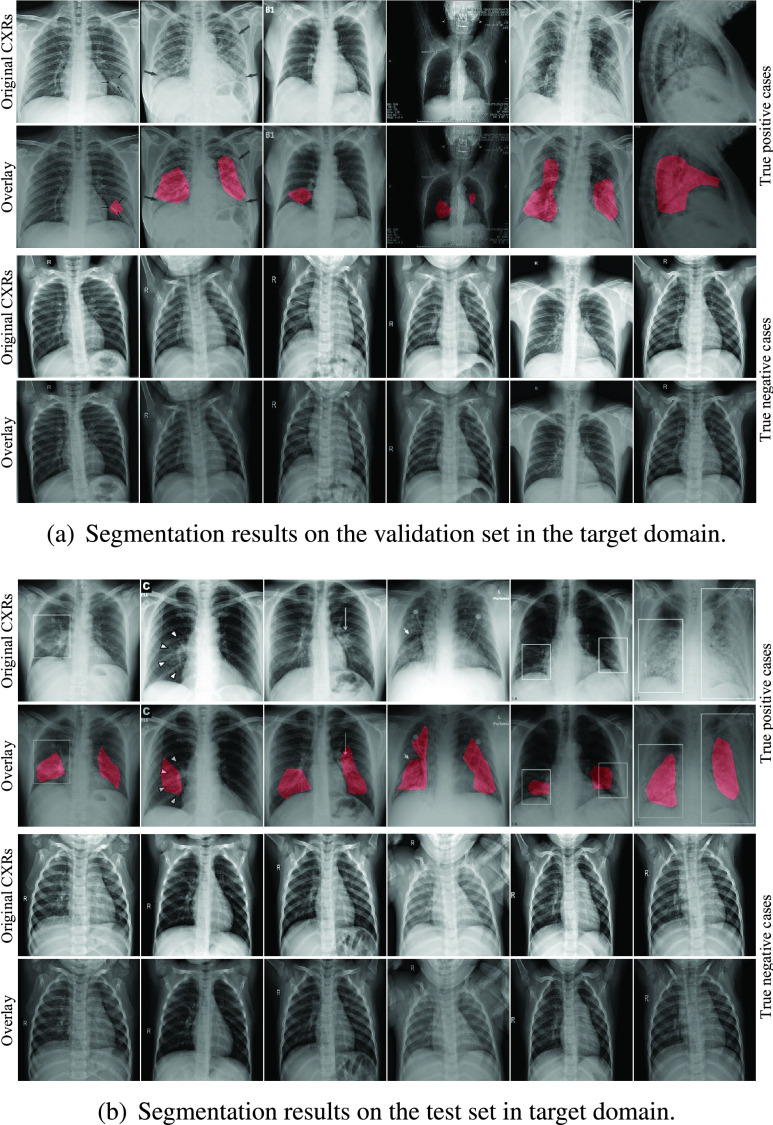
COVID-19 infection segmentation results of the infection-aware DRRs with $w_{2}=3.0$ and $T^{2}=0.20$ on the validation and test sets in target domain. The red overlay is used to indicate the infected regions.

**FIGURE 16.**
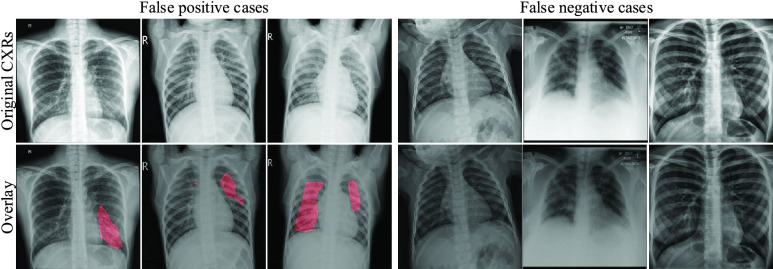
Failure cases of the segmentation results of the infection-aware DRRs with $w_{2}=3.0$ and $T^{2}=0.20$ on the validation and test sets. The red overlay is used to indicate the infected regions.

We specifically use the case of infection-aware DRRs with $w_{2}=3.0$ and $T^{2}=0.20$ as an example to show the COVID-19 infection segmentation results. The segmentation scores, including accuracy, AUC, and F1-score, on the validation and test sets are (0.919, 0.977, 0.910) and (0.956, 0.980, 0.959) respectively as listed in Table 7, 8, 9, 13, 14, and 15 of the appendix. Next, we visualize the infection segmentation results from the first fold. The confusion matrices of the segmentation results on the corresponding validation and test sets are shown in Fig. 14. We visualize several true positive and true negative cases in Fig. 15. Compared with previous studies that highlight the infected regions roughly by leveraging the interpretability of deep classification models, our segmentation model trained on the infection-aware DRRs is able to segment the infected regions in CXRs directly and accurately. Besides, we present several failure (false positive and false negative) cases in Fig. 16.

#### 4) Estimating the Detection Limit of X-Ray Imaging in Detecting COVID-19 Infection

**FIGURE 17.**
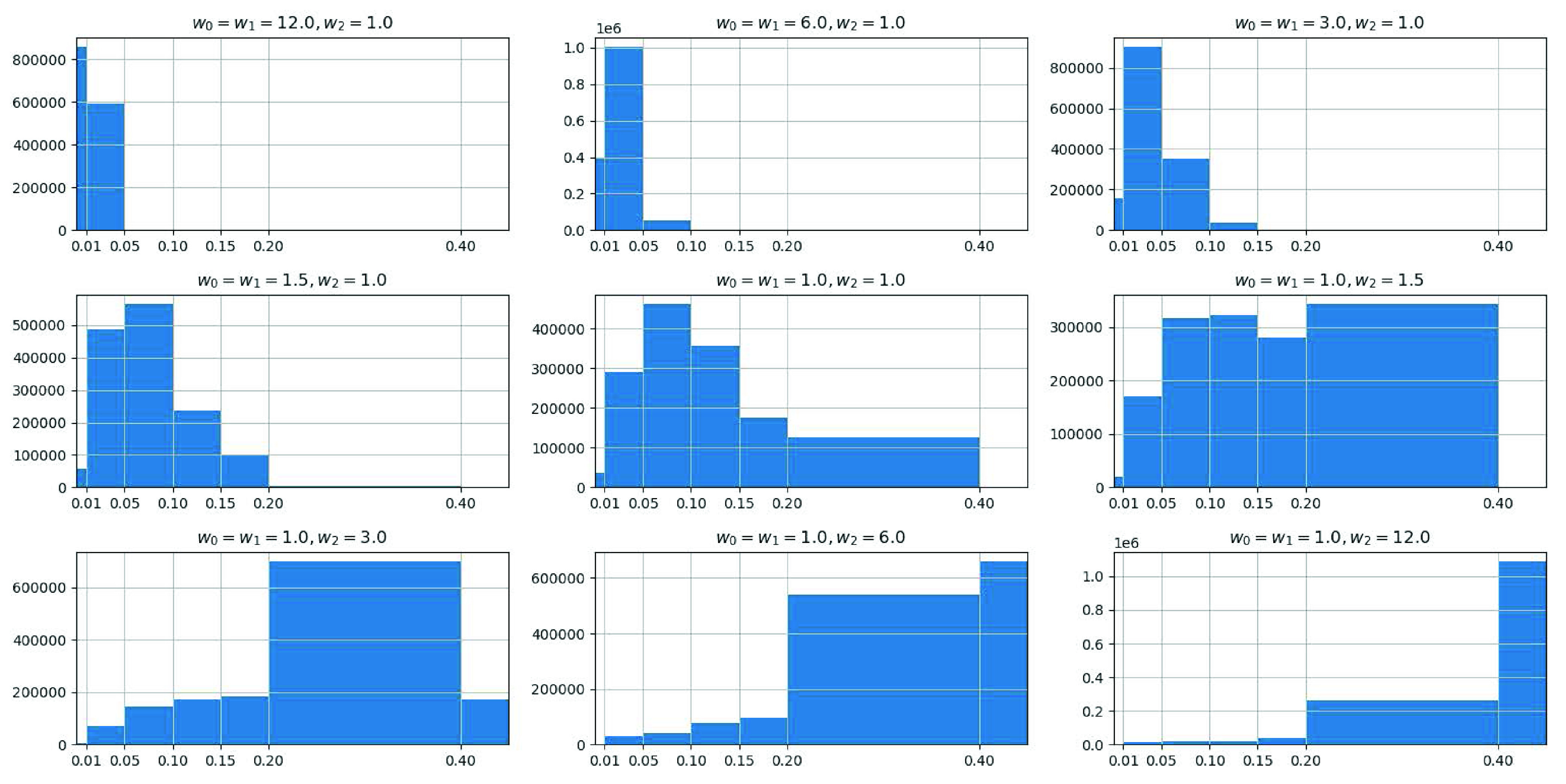
Histograms of infected voxel contribution rates of the pixels in infection-aware DRRs.

As mentioned earlier, CXRs are generally considered less sensitive than 3D CT scans [7]. It may happen that CT examination detects an abnormality, whereas the X-ray screening on the same patient reports no findings. The significance level of radiological signs of COVID-19 infection in X-ray images depends on the severity of COVID-19 infection, which is typically assessed by the percent volume of the lung that is infected by COVID-19 (PIV for short). Only when the PIV reaches a certain level (i.e., detection limit), X-ray imaging can effectively detect COVID-19 infection. Therefore, we propose to leverage our infection-aware DRR generator by searching the best parameters $(w_{0},w_{1}, w_{2})$ and $T^{2}$ to estimate such detection limit. The insight behind this estimation method is that the DRRs, generated by using the best parameters, have the smallest gap with real positive X-ray images, and thus will achieve the highest classification and segmentation scores no matter whether the domain adaptation module is appied or not. Accordingly, we average the corresponding items in Table 4–27 of the appendix to obtain the total average (T-Avg.) scores of 63 training sets to search for the best parameters. Meanwhile, we compute the equivalent average PIV (EAPIV) of the 10 CT cases that are used to generate infection-aware DRRs. As can be seen from Table 2, the peak of T-Avg. scores at each row in Table 2 appears consistently at $w_{2}=3.0$, and the corresponding EAPIV is 19.43% ± 16.29% (Mean ± Standard Deviation). When the EAPIV is less than 19.43% ($w_{2}=3.0$), e.g., 11.77% ($w_{2}=1.5$) and 8.51% ($w_{2}=1.0$), the corresponding T-Avg. scores drop below 81.0% no matter what the contribution threshold of infected voxels (CTIV) $T^{2}$ is. Such results imply that DRRs generated from a CT case whose PIV is less than 19.43% cannot be easily distinguished from normal DRRs. Therefore, we conclude that the detection limit of X-ray imaging, measured by the percent volume of the lung that is infected by COVID-19, is 19.43% ± 16.29%. Moreover, to examine the function of CTIV, we plot the histograms of the CRIV of the pixels in infection-aware DRRs in Fig. 17. Note that only the pixels whose corresponding X-rays pass through the infected voxels of CT volume are counted. These histograms show the effectiveness of our infection-aware DRR generator in changing the distribution of CRIV of the pixels in infection-aware DRRs. For the best parameters $w_{0,1}=1.0$ and $w_{3}=3.0$ (the 8-th column of Table 2), the peak of T-Avg. scores (87.9%) appears at $T^{2}=0.20$. Increasing the CTIV $T^{2}$ from 0.2 to 0.4 makes a large numberof pixels (more than 600,000) whose CRIVs are between 0.2 and 0.4 be treated as negative pixels, thus leading to a significant drop in T-Avg. score (-6.9%$\downarrow $). Meanwhile, decreasing the CTIV $T^{2}$ from 0.2 to 0.15 makes the pixels (less than 200,000) whose CRIVs are between 0.15 and 0.2 be treated as positive pixels, thus leading to a minor drop in T-Avg. score (-0.9%$\downarrow $). Therefore, we conclude that the estimated lower bound of CRIV for significant radiological signs of COVID-19 infection in DRRs is 20.0%. It means that the pixels whose CRIVs are lower than 20.0% cannot be easily distinguished from the pixels of the lungs in CXRs.

#### 5) Evaluation of Segmentation Performance on 10 CXRs

We simply use the 4-fold cross validation models trained by using our domain adaptation module to segment the 10 CXRs. The average DSC scores of these segmentation results are reported in Table 3. As can be seen, our infection-aware DRRs ($w_{2}=3.0$ or $w_{2}=6.0$) have achieved an average DSC score of $\sim 40$%, which are much better (>20%) than the standard DRRs. Besides, we also observe the same pattern as in Table 2: the peak of average DSC scores of our infection-aware DRRs appears at $w_{2}=3.0$. It provides more evidence for confirming the validity of the estimated detection limit of X-ray imaging in detecting COVID-19 infection.

## V. Limitations of the Study

This study still has a variety of limitations. Firstly, the synthetic annotation masks of infected regions for DRRs are only investigated experimentally, and are not evaluated by the radiologists. The expert annotations of infected regions for DRRs may provide evidence to determine more accurate contribution threshold of infected voxels. Secondly, this study uses publicly available CT and CXR datasets from various sources. The variability in expert annotations of CT scans and CXRs is not assessed, which may introduce implicit biases to the training and evaluation. Thirdly, the evaluation for the segmentation performance of our method in this study is incomprehensive due to the lack of sufficient CXRs with pixel-level annotations of infected regions. The evaluation results on ten CXRs may be biased and unable to give guidance to optimize our method. A comparison between learning infection segmentation from CXRs straightway and learning from DRRs may provide key insights on how to realize high-quality automated COVID-19 infection segmentation on CXRs efficiently. Lastly, this study uses only ten COVID-19 positive CT cases for synthesizing DRRs. The performance of our DRR4Covid and the validity of the estimated detection limit of X-ray imaging in detecting COVID-19 infection can be improved by involving more CT scans from patients in various stages of COVID-19 infection. Nevertheless, the findings of this article provide promising results that encourage the use of DRR4Covid for learning automated COVID-19 infection segmentation on CXRs from DRRs without the need for any expert annotations of CXRs.

## VI. Conclusion

We propose a novel approach, called DRR4Covid, to learn automated COVID-19 infection segmentation on CXRs from DRRs. DRR4Covid consists of three key components, including an infection-aware DRR generator, a classification and segmentation network, and a domain adaptation module. The infection-aware DRR generator is able to produce DRRs with adjustable strength of radiological signs of COVID-19 infection, and generate pixel-level infection annotations that match the DRRs precisely, thus enabling deep segmentation networks to be trained directly for automated infection segmentation. The domain adaptation module is introduced to reduce the domain discrepancy between DRRs and CXRs by training networks on unlabeled real CXRs and labeled DRRs together. We provide a simple but effective implementation of DRR4Covid by using a domain adaptation module based on Maximum Mean Discrepancy (MMD), and a FCN-based network with a classification header and a segmentation header. Extensive experiment results have confirmed the efficacy of our method; specifically, without using any annotations of real CXRs, our network has achieved a classification score of (Accuracy: 0.949, AUC: 0.987, F1-score: 0.947) and a segmentation score of (Accuracy: 0.956, AUC: 0.980, F1-score: 0.955) on a test set with 558 normal cases and 558 positive cases. Besides, we estimate the detection limit of X-ray imaging in detecting COVID-19 infection by adjusting the strength of radiological signs of COVID-19 infection in synthetic DRRs. The estimated detection limit, measured by the percent volume of the lung that is infected by COVID-19, is 19.43% ± 16.29%, and the estimated lower bound of the contribution rate of infected voxels is 20.0% for significant radiological signs of COVID-19 infection.

To our best knowledge, this is the first attempt to realize the automated COVID-19 infection segmentation base on CXRs by using the labeled DRRs that are generated from Chest CT scans. Future work can be carried out by extending the DRR4Covid to DRR4Lesion to enable multiple lung lesion segmentation on CXRs.

## Appendix More Experimental Results

See Tables 4–27.
